# Stable radical anions generated from a porous perylenediimide metal-organic framework for boosting near-infrared photothermal conversion

**DOI:** 10.1038/s41467-019-08434-4

**Published:** 2019-02-15

**Authors:** Baozhong Lü, Yifa Chen, Pengyu Li, Bo Wang, Klaus Müllen, Meizhen Yin

**Affiliations:** 1 State Key Laboratory of Chemical Resource Engineering, Beijing Advanced Innovation Center for Soft Matter Science and Engineering, Beijing Laboratory of Biomedical Materials, Beijing University of Chemical Technology, 100029 Beijing, P. R. China; 2 Key Laboratory of Cluster Science, Ministry of Education, School of Chemistry and Chemical Engineering, Beijing Institute of Technology, 100081 Beijing, P. R. China; 3 Max Planck Institute for Polymer Research, Institute of Physical Chemistry, Johannes Gutenberg University Mainz, Duesbergweg 10–14, 55128 Mainz, Germany

## Abstract

Radical anions of electron-deficient systems are widely used, but are easily reoxidized upon exposure to air. Therefore, the stabilization of radical anions under ambient conditions is of great significance, but still remains a scientific challenge. Herein, perylenediimide is employed to prepare a crystalline metal-organic framework for stabilizing radical anions without extensive chemical modification. The porous, three-dimensional framework of perylenediimide can trap electron donors such as amine vapors and produce radical anions in-situ through photo-induced electron transfer. The radical anions are protected against quenching by shielding effect in air and remain unobstructed in air for at least a month. Because of the high yield and stability of the radical anions, which are the basis for near-infrared photothermal conversion, the framework shows high near-infrared photothermal conversion efficiency (η = 52.3%). The work provides an efficient and simple method towards ambient stable radical anions and affords a promising material for photothermal therapy.

## Abstract (web summary)

Perylenediimide (PDI) radical anions show promise as photothermal agents for phototherapy, but are typically very unstable. Here the authors fabricate a PDI-based metal-organic framework where the PDI units become stable radical anions upon incorporation of amine vapors.

## Introduction

Near-infrared (NIR) photothermal materials convert absorbed NIR light into thermal energy by inhibiting the radiative relaxation of materials^1,2^. Because of the deep penetration ability and dark field viewing properties of NIR light, the NIR photothermal effect has been of interest in applications such as NIR photothermal ablation^3^, NIR laser-assisted photothermal therapy^4^, and night vision sensors^5^. Conventional organic NIR-absorbing molecules may not only require complex synthesis procedures but also easily suffer from photobleaching under light irradiation. These drawbacks lead to high costs and a risk of performance decay in photothermal processes. Thus, it is essential to devise an organic photothermal agent with stable photothermal capacity.

Perylenediimides (PDIs) are an outstanding class of organic dye molecules that have been utilized for nearly a century^6–8^. Owing to the ease of reduction into delocalized radical anions (RAs)^9^, these versatile organic molecules are of interest in photocatalysis^10^, n-channel transistor fabrication^11^, and photoconduction^12^. PDI RAs (PDI^•−^) display a typical red-shift absorption towards the NIR region. Such useful NIR absorbers play an important role as photothermal agents for photothermal therapy. Photothermal therapy has significant advantages over chemotherapy and radiotherapy due to its superior local efficacy and fewer systemic side effects^2–4^. PDI^•−^ have already been used to regulate the balance of bacterial flora^13–15^. However, these applications, especially NIR photothermal conversion, are adversely affected by the instability of the RAs under ambient conditions^16–26^. The formation and use of ambient stable PDI^•−^ thus remain of prime importance. Many efforts have been devoted to improving the stability of RAs, such as covalently modifying PDI cores and incorporating RAs into films or supramolecular assemblies^13,20–23^. These efforts are often demanding from both a synthesis and a processing point of view. Except for an imidazolium-modified zwitterionic PDI, the RAs are rapidly reoxidized upon exposure to air^20^. Functionalized PDI materials without extensive chemical modification, but yielding stable RAs under ambient conditions, would be highly desirable.

Metal-organic frameworks (MOFs) are a class of robust, porous, crystalline hybrid materials that have received much attention due to their myriad of properties and simple synthesis^27–31^. It is indeed possible to obtain rationally designed functional MOFs by selecting various metal ions and organic ligands or by adjusting the synthesis conditions. Among other functions, three-dimensional (3D) porous MOFs can serve as stable cages^32–34^. However, the known MOFs do not on their own produce RAs because of the lack of strongly electron-deficient aromatic ligands. We reasoned that cages composed of PDI ligands could produce RAs in situ with improved stability due to a pronounced shielding effect. PDI-based 3D MOFs have not been reported because (1) PDIs with coordinating carboxyl or pyridyl functions exhibit poor solubility in common solvents and (2) PDIs tend to form one-dimensional or two-dimensional assemblies with close π–π stacking, which inhibits porosity^7,35–38^.

**Fig. 1:**
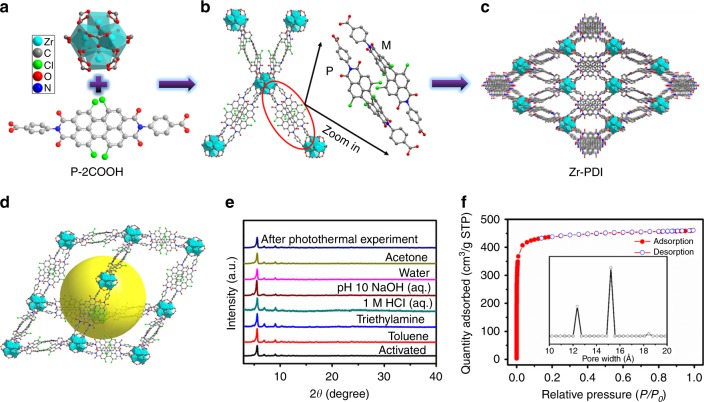
Synthesis of 3D porous Zr-PDI. **a** Structures of Zr-cluster and P-2COOH. **b** Connection mode of Zr-cluster (the Zr-cluster is fully coordinated by 12 carboxylate units) and molecular arrangement of chiral P-2COOH. **c** a-Axis crystal structure of Zr-PDI. **d** Single distorted adamantanoid cage (the distorted cage is similar to adamantane) in Zr-PDI. Hydrogen atoms have been omitted for clarity. **e** Results of stability tests of Zr-PDI incubated under various conditions for 24 h. Zr-PDI powder is washed with acetone and dried under vacuum at 120 °C prior to measurement. **f** N_2_ sorption isotherm at 77 K and corresponding pore size distribution

To elucidate this concept, we report a PDI-based 3D MOF, Zr-PDI, composed of a *N*,*N*′-di-(4-benzoic acid)-1,2,6,7-tetrachloroperylene-3,4,9,10-tetracarboxylic acid diimide (P-2COOH) ligand and Zr_6_(μ_3_-O)_4_(μ_3_-OH)_4_ clusters (Fig. 1). The non-planar structure of P-2COOH enhances its solubility in polar solvents, thus allowing the successful solvothermal synthesis of Zr-PDI. Zr^4+^ is chosen because the reported Zr-based MOFs exhibit remarkable stability and high porosity^29^. Zr-PDI forms a fascinating 3D porous network and can serve as cage to trap electron donors such as organic amines to furnish RAs (Zr-PDI^•−^) in situ by photo-induced electron transfer (PET). Being confined in a framework, Zr-PDI^•−^ has superior stability in air and remains intact for at least a month. To the best of our knowledge, Zr-PDI^•−^ is the first example of isolated RAs which are stable at ambient conditions without modification. A similar stability cannot be obtained by using the ligand P-2COOH^•−^ under the same conditions. Zr-PDI^•−^, with NIR absorbance, in addition to high yield and stability, shows an exceptionally high NIR photothermal conversion efficiency (η = 52.3%) and good recyclability. In comparison, P-2COOH^•−^ shows poor NIR photothermal conversion efficiency and recyclability.

## Results and discussion

### Framework formation and structural characterization

The solvothermal reaction of P-2COOH (Supplementary Figs 1 and 2) and ZrCl_4_ in dimethyl formamide (DMF) in the presence of acetic acid as a modulator (a coordination modulator controls coordination kinetics and the resultant crystal size) produced Zr-PDI in a yield of 58%. Single-crystal analysis revealed that Zr-PDI crystallized in the tetragonal space group I41/a (Supplementary Table 1). In the Zr_6_(μ_3_-O)_4_(μ_3_-OH)_4_ octahedral cluster formed, the six vertices of the octahedron are occupied by Zr(IV) centers and the eight triangular faces are alternatively capped by four μ_3_-OH and four μ_3_-O groups, as shown in Fig. 1a. The Zr_6_(μ_3_-O)_4_(μ_3_-OH)_4_ cluster is fully coordinated by 12 carboxylate units (i.e. 8 P-2COOH ligands and 4 acetic acid molecules) (Fig. 1b). From a topological point of view, a 3D framework originates from a highly symmetric Zr_6_(μ_3_-O)_4_(μ_3_-OH)_4_-(CO_2_)_12_ secondary building unit and organic ligands (Fig. 1c). Owing to the electrostatic repulsion and steric hindrance between the chloro substituents, the central six-membered ring of P-2COOH is highly twisted with a dihedral angle of 38.3° (Supplementary Fig. 3) associated with the bay carbon atoms C11-C12-C20-C21. This twist also induces the axial chirality of PDIs, which can be observed in the crystalline state (Fig. 1b)^39^. Figure 1b shows two P-2COOH ligands with opposite chirality, which serve as a bridge connecting two Zr_6_(μ_3_-O)_4_(μ_3_-OH)_4_ clusters.

MOFs incorporating extended linkers such as P-2COOH (length ~24 Å) are often unstable and undergo pore collapse upon solvent removal. Figure 1d displays a huge distorted adamantanoid cage in Zr-PDI. While minimizing the large cavities in the adamantanoid cages, a normal five-fold non-connected interpenetration is formed, as illustrated in Supplementary Fig. 4. Upon inspection of the powder X-ray diffraction (PXRD) patterns, it is clear that Zr-PDI is highly crystalline, consistent with the simulated pattern (Supplementary Fig. 5). Scanning electron microscopy (SEM) images reveal that the bulk microcrystalline powder consists of cubic crystallites ~2 μm in length and with a smooth surface (Supplementary Figs 6 and 7). Stability tests were performed by incubating the as-synthesized Zr-PDI under various conditions, and the results are shown in Fig. 1e. Identical PXRD patterns were obtained before and after incubation, confirming the stability of the framework. N_2_ sorption analysis of Zr-PDI gave a Brunauer−Emmett−Teller (BET) surface area of 1330 m²/g and a total pore volume of 0.71 cm^3^/g by a volumetric method, comparable to those of UiO-66 (ref. ^29^). Type Ι isotherms are observed for Zr-PDI (Fig. 1f), indicating dominating microporous adsorption. Indeed, Zr-PDI exhibits mainly two types of pores with sizes of ∼12 and ∼15 Å.

### Optical properties and formation of RAs

**Fig. 2:**
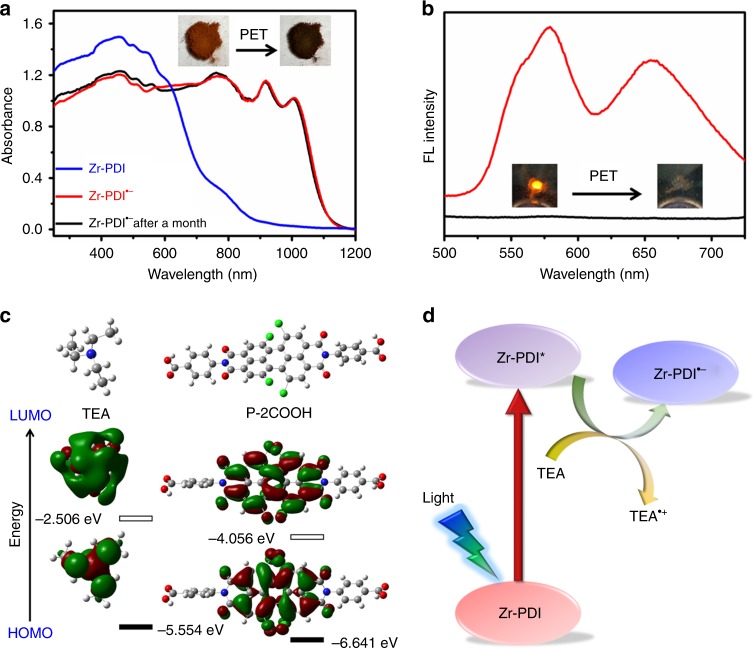
PET between triethylamine (TEA) and Zr-PDI. **a** UV–Vis–NIR absorption and **b** fluorescence spectra of Zr-PDI before (red line) and after (black line) PET. The insets show the photographs of color and fluorescence changes. **c** Energy diagrams optimized by using the B3LYP function and the 6-31G (d) basis set and pictorial representations of HOMO and LUMO orbitals of TEA and P-2COOH in their ground states. **d** Schematic diagram of the PET process between TEA and Zr-PDI, excited state Zr-PDI^*^ is reductively quenched by TEA to afford Zr-PDI^•−^

The arrangement of P-2COOH molecules within the framework makes it a remarkable model to study the optical properties of Zr-PDI (Supplementary Figs 8–12). In the UV–Vis–NIR absorption spectrum in Fig. 2a, Zr-PDI exhibits mainly three PDI absorptions in the 450–560 nm range, which are similar to the absorptions of alkyl-substituted PDI thin film^40^. Zr-PDI also gives a red-shift of the broad absorption in the visible region from that of the corresponding monomer in DMF, and this shift is due to a combination of excitonic coupling and charge transfer interactions between the π-stacked PDI chromophores (Supplementary Fig. 13)^41^. Upon irradiation with blue light (455 nm) in the presence of TEA vapor as an electron donor, the red Zr-PDI powder turned black in 10 min. The absorption spectrum of Zr-PDI^•−^ displays three new peaks at 778, 917, and 1008 nm, which are characteristic peaks of PDI^•−^ produced by PET^9,20^. The new peaks could be assigned to the D_0_ → Dn transition with a complex electronic vibration. In comparison, when treated similarly, the P-2COOH crystalline powder (Supplementary Fig. 14) produced only a low yield of P-2COOH^•−^, as presented in its absorption spectrum (Supplementary Fig. 15)^13^.

Fluorescence quenching of Zr-PDI^•−^ confirmed the formation of RAs (Fig. 2b). There are two fluorescence peaks of Zr-PDI: one is a monomer emission peak at around 578 nm and the other is a broad peak at around 657 nm (Supplementary Figs 16–18). The latter can be regarded as an excimer emission from the perylene groups^42^. All experiments and calculations support the proposed PET process demonstrated in Fig. 2d: the excited state Zr-PDI^*^ is reductively quenched by TEA to afford Zr-PDI^•−^ and the radical cation TEA^•+^.

**Fig. 3:**
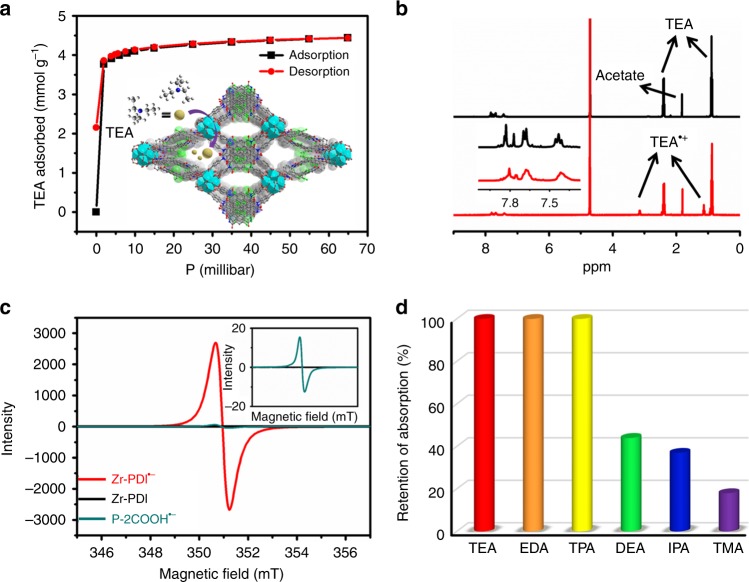
Characterizations of Zr-PDI^•−^. **a** TEA adsorption and desorption of activated Zr-PDI; inset shows TEA vapor in Zr-PDI channel. **b** ^1^H NMR spectra of TEA-loaded Zr-PDI before (black line) and after (red line) irradiation; inset shows the peaks of PDI. **c** EPR spectra of solid Zr-PDI^•−^ and P-2COOH^•−^; inset shows the magnified spectrum of P-2COOH^•−^. **d** Stability of Zr-PDI^•−^ loaded with different amines under ambient conditions after a week. The ordinate represents the retention rates of the absorption peak at 1008 nm. TEA triethylamine, EDA ethylenediamine, TPA tripropylamine, DEA diethylamine, IPA isopropylamine, TMA trimethylamine

The facile production of Zr-PDI^•−^ provides evidence that Zr-PDI has an outstanding ability to adsorb TEA. To calculate the exact sorption capacity of TEA, we measured the TEA sorption test of activated Zr-PDI (MOF without solvent in the pore) sample at 298 K (Fig. 3a). Zr-PDI gave type I isotherms, with steep uptakes at low absolute pressures. The total TEA uptake capacity at 60 millibars was 4.44 mmol g^−^^1^ (or 0.45 g g^−1^) for Zr-PDI (Supplementary Fig. 19). Considering the size and molecular weight of TEA, Zr-PDI exhibits high amine capacities with an equilibrium uptake of 9.06 molecules of TEA per P-2COOH molecule^43^. The excellent sorption capacity ensures the efficient PET reaction between TEA and Zr-PDI. The TPA sorption was also measured (Supplementary Fig. 20), the sorption isotherm of TPA being similar to that of TEA. The desorption and adsorption curves nearly coincide, indicating that there is no specific interaction between MOF and amines. The incorporation of TEA inside the framework was then probed by ^1^H nuclear magnetic resonance (NMR) spectroscopy. After adsorbing TEA vapor, Zr-PDI was dissolved in NaOH/D2O solution, and two additional chemical shifts corresponding to the proton signals of -CH_2_- and -CH_3_ of TEA are observed in Fig. 3b. After the TEA-loaded Zr-PDI was irradiated with 455 nm light, two new peaks belonging to TEA^•+^ emerged. Besides, only broadened resonances belonging to PDI after irradiation were detected due to the paramagnetic nature of Zr-PDI^•−^.

Electron paramagnetic resonance (EPR) spectroscopy was carried out for Zr-PDI samples before and after PET to support the formation of PDI^•−^. As presented in Fig. 3c, Zr-PDI, after treatment with TEA and irradiation, displays a typical EPR signal, confirming the existence of free radicals. The intense resonance has no resolved hyperfine coupling, which is in accordance with literature data^20^. Zr-PDI^•−^ and P-2COOH^•−^ have the same *g* value of 2.0046 (Supplementary Figs 21 and 22), pointing toward the formation of PDI^•−^^14^. It has been reported that the integration of the EPR signal is proportional to the yield of RAs^13^. The integration of the EPR signal was calculated to be 21.7 for P-2COOH^•−^ and 3639.1 for Zr-PDI^•−^. Therefore, the yield of Zr-PDI^•−^ was about 168 times that of P-2COOH^•−^, consistent with the above UV–Vis–NIR data.

### RAs stable under ambient conditions

Most anionic PDI species are readily reoxidized upon exposure to air^16–26^. To evaluate the stability of Zr-PDI^•−^, we kept TEA-loaded Zr-PDI^•−^ under ambient conditions (open air and laboratory light) or at different temperatures. Encouragingly, under ambient conditions, Zr-PDI^•−^ remained almost unaffected for over a month (Fig. 2a), while the RAs produced by P-2COOH or P-2COOH/ZrCl_4_ mixtures disappeared completely in 12 h (Supplementary Fig. 23). Furthermore, Zr-PDI^•−^ was rather stable (detected at 778 nm in UV–Vis–NIR spectra) at high temperatures (retention rates of 89% at 120 °C and 76% even at 180 °C) after 1 h (Supplementary Figs 24 and 25), while under the same conditions, P-2COOH^•−^ was completely destroyed by oxidation and high temperatures (Supplementary Fig. 26). The porous framework of Zr-PDI appears to stabilize RAs under ambient conditions and at high temperatures. The PET process leading to Zr-PDI^•−^ crystalline powder was performed under ambient conditions and required neither protective measures nor low temperatures often necessary for the crystallization of radical ions. Direct formation of Zr-PDI^•−^ by P-2COOH^•−^ was also attempted, but failed to provide Zr-PDI^•−^. Obviously, the solvothermal reaction requires high temperature and modulators, so that the RAs will be quenched.

There are three state-of-the-art ambient stable aromatic diimide RAs obtained by covalent modification of aromatic cores^17,20,44^. The displayed stability of Zr-PDI^•−^ under ambient conditions is not inferior to that reported in previous studies^17,20^, but without any extra modifications in the tetrachlorinated aromatic core. The present result calls for a closer look on the improved stability of Zr-PDI^•−^. The PET processes between Zr-PDI and different amines were investigated (Fig. 3d). Zr-PDI^•−^ is not supposed to form when large amines (~15 Å) are used (Supplementary Fig. 27), simply because they cannot enter the pores of Zr-PDI. Some amine-loaded Zr-PDIs^•−^ are presented in Fig. 3f. DEA, EDA, and TEA have comparable sizes of 6.8, 5.4, and 6.6 Å (Supplementary Fig. 28), respectively, which are much smaller than the pore sizes of Zr-PDI (~12 and ~15 Å). Nevertheless, the three amine-loaded Zr-PDIs^•−^ display large differences in stability (Supplementary Fig. 29), so the size of the amine used is irrelevant to the stability of RAs. After TEA-loaded Zr-PDI^•−^ were heated for 120 °C, some TEA leaked from Zr-PDI^•−^. But, the amount of TEA^•+^ was constant, and the stability of the residual Zr-PDI^•−^ remained almost unchanged for another month under ambient conditions (Supplementary Figs 30 and 31). Thus, the loading of the amine used is irrelevant to the stability of RAs. By comparing amines with different boiling points, we found that the higher the boiling point of the amine used the higher the stability of the Zr-PDI^•−^, as further confirmed by ^1^H NMR (Supplementary Table 2 and Supplementary Figs 32 and 34). Amines with low boiling points have the tendency to leak from Zr-PDI^•−^ under ambient conditions, resulting in the instability of RAs. However, amines with high boiling points enable to stay in Zr-PDI under ambient conditions and even at high temperatures. The interpenetrating nature of Zr-PDI leads to a highly porous network with small cages^43^, so it is most likely that high boiling point amines together with amine radical cations are trapped in these cages to form stable adducts. Also, the reduced π–π stacking of PDIs in the framework prevents RAs to dimerize and quench^13^. Therefore, one can not only improve the yield of RAs but achieve a pronounced stabilization by confining them in the MOF cages.

### Photothermal conversion of Zr-PDI^•−^

**Fig. 4:**
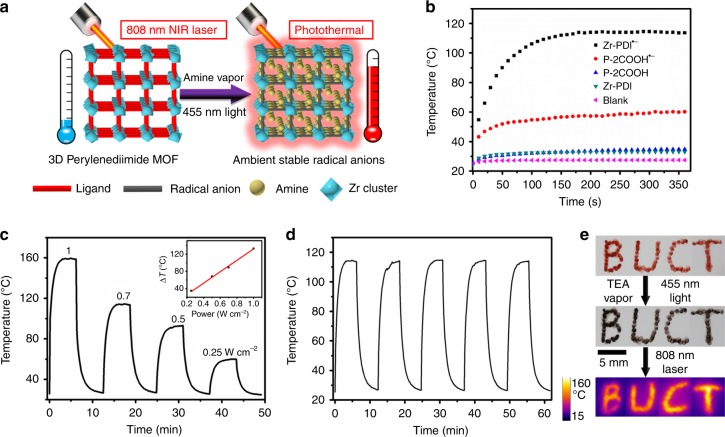
Photothermal conversion performance of Zr-PDI^•−^. **a** Illustration of the colored PDI^•−^ formation and photothermal conversion of Zr-PDI^•−^. **b** Photothermal conversion curves of Zr-PDI^•−^ film on quartz glass under laser irradiation (808 nm, 0.7 W cm^−^^2^). **c** Temperature rises of Zr-PDI^•−^ at different NIR laser intensities. Inset shows average temperature rise (Δ*T*) as a function of NIR laser power. **d** Photothermal cycling curve of Zr-PDI^•−^ film (808 nm, 0.7 W cm^−^^2^). **e** IR camera images of Zr-PDI-patterned letters BUCT (808 nm, 0.7 W cm^−2^)

Zr-PDI^•−^ with NIR absorbance can be produced facilely and has extraordinary stability. To further verify and use this stability, we utilized Zr-PDI^•−^ as a NIR photothermal material (Fig. 4a). Under irradiation with NIR laser (808 nm, 0.7 W cm^−^^2^), the temperature of Zr-PDI^•−^ powder reached 160 °C in 10 s (Supplementary Movie 1). Then to accurately measure the photothermal conversion, Zr-PDI^•−^ powder was fixed on a piece of quartz glass to form a uniform film. Under irradiation with 808 nm laser light (0.7 W cm^2^), the temperature of the quartz glass was sharply increased to as high as 114 °C, a temperature rise of over 89 °C within 200 s (Supplementary Movie 2). In comparison, with NIR laser irradiation, the temperature of a piece of blank quartz glass rose only 2.4 °C, and the temperature of a piece of quartz glass coated with a film of Zr-PDI rose only 9 °C due to the lack of NIR absorbance (Fig. 4b). These results imply that Zr-PDI^•−^ has fascinating photothermal conversion properties, which is comparable to recently reported photothermal conversions of MOFs using UV–Vis light^45^. Lower temperature increases were recorded for P-2COOH^•−^ because of the low yield of RAs, indicating that it cannot serve as a photothermal conversion material. The cooling curve of Zr-PDI^•−^ film, from which the conversion efficiency can be calculated (details are shown in Supplementary Note 1), is presented in Supplementary Fig. 35. An exceptionally high conversion efficiency (52.3%) was obtained, much higher than most reported photothermal materials such as Au nanorods (21.0%), organic cocrystals (18.8%), and selenophene-derived polymer films (40%) (Supplementary Table 3)^46,47^. Fig. 4c demonstrates that the photothermal effect is linearly dependent on the NIR laser power from 0.25 to 1 W cm^−2^, an indication of a thermal control performance. Correspondingly, there is no significant light emission in Zr-PDI^•−^ (Supplementary Fig. 36), demonstrating the dominant non-radiative transition, in agreement with the high photothermal conversion. Notably, the PXRD pattern of Zr-PDI after laser irradiation is in agreement with the powder data (Fig. 2e), indicating that the crystal structure of Zr-PDI remains intact upon laser irradiation. Zr-PDI after photothermal conversion has a slight decrease in the N_2_ adsorption, because of the residual TEA^•+^ in the framework (Supplementary Fig. 37). The cycling test results (Fig. 4c) also demonstrate the high stability of Zr-PDI^•−^ at high temperatures, which was attributed to the effective NIR absorbance of TEA, TPA, EDA-loaded Zr-PDI^•−^ (Supplementary Figs 24 and 25). However, P-2COOH^•−^ was unstable at high temperatures and showed inferior cycle test results (Supplementary Fig. 38). The high photothermal conversion of Zr-PDI^•−^ convincingly displays the potential of the RAs in photothermal imaging, an illustration of which is shown in Fig. 4e. Under irradiation with NIR light, photothermal materials reveal a significant temperature increase, which can be captured with an IR thermal camera. We then fabricated the letter pattern BUCT with Zr-PDI^•−^ powder. Under irradiation with NIR light (808 nm, 0.7 W cm^−^^2^), the powder pattern became imageable with a high resolution. The temperature increase of Zr-PDI^•−^ is large enough in bio-imaging and biomedical applications such as photothermal therapy^48–51^. Also, the PET process under formation of Zr-PDI^•−^ can be initiated by medically active dopamine (Supplementary Figs 39 and 40). To the best of our knowledge, this is the first direct NIR photothermal conversion of a MOF without extra chemical modification by using RAs^52–54^.

In summary, a PDI-based 3D MOF (Zr-PDI) with ultrastable RAs provides a unique platform for NIR photothermal conversion. The suitable pores allow electron donor amine vapors to occupy the cages of Zr-PDI. Upon irradiation with blue light (455 nm), black Zr-PDI^•−^ with NIR absorbance can be formed through PET. The produced RAs, which are in the Zr-PDI cages, can stay unobstructed under ambient conditions for at least a month. So a strategy to stabilize PDI^•−^ without complicated design and tedious synthesis was discovered. Under 808 nm laser irradiation, the temperature of the Zr-PDI^•−^ sharply increases, which has a superior photothermal conversion efficiency of 52.3% due to non-radiative pathway. With post-synthesis modifications, this MOF material, with outstanding stability, have great potential in biomedical applications such as bio-imaging and photothermal therapy, and the excited RAs may be useful as photoelectrochemical catalysts^5,35^. This work not only provides an efficient method towards ambient stable RAs, but also gives insights into the development of novel PDI materials.

## Methods

### General procedure for Zr-PDI preparation

A mixture of P-2COOH (13.8 mg, 0.018 mmol), ZrCl_4_ (4.3 mg, 0.018 mmol) was dissolved in 10 mL DMF and 0.45 mL acetic acid. The resulting mixture was heated in a 20 mL bottle with cap at 90 °C for 3 days, and then allowed to cool slowly to room temperature. Red crystals were collected and washed with chloroform for three times and dried at vacuum. Yield: 58% (based on P-2COOH). Anal. Calcd (%) for Zr-PDI (C_80_H_36_C_l8_N_4_O_27_Zr_3_): C, 47.01; H, 1.8; N, 2.74. Found: C, 48.53; H, 2.28; N, 2.69.

### Gas adsorption isotherms of Zr-PDI

Nitrogen adsorption isotherms were measured by a volumetric method using a Micromeritics ASAP 2460 gas sorption analyzer. A sample of ca. 111.6 mg Zr-PDI pre-activated at 100 °C to remove all residual solvent. Nitrogen isotherms were measured using UHP grade nitrogen. All nitrogen analyses were performed using a liquid nitrogen bath at −195.8 °C. TEA adsorption isotherms were measured by a gravimetric method using an IGA 002 vapor sorption analyzer. A sample of ca. 33.8 mg Zr-PDI, pre-activated at 100 °C to remove all residual solvent. TEA adsorption analyses were performed using a water bath at 25 °C and under vapor pressure of TEA.

### Photothermal conversion properties measurement

One milligram Zr-PDI powders were dispersed in 0.1 mL acetone; the mixture was drop-cast carefully on a quartz glass (0.8 × 0.8 cm, 320 mg) to form a uniform film. The film was then treated with TEA vapor and blue light (455 nm) for 10 min to produce Zr-PDI^•−^. The 808 nm laser beam was irradiated at a power density from 0.25 to 1 W cm^−2^. The temperature was monitored every 10 s by a Fluke (Ti400) thermal imaging camera. Photothermal conversion efficiency of the Zr-PDI^•−^ was calculated by recording the change in the temperature of the quartz glass coated with Zr-PDI powders as a function of time under continuous irradiation of a 808 nm laser (0.7 W cm^−2^) until the glass reached a steady-state temperature.

## Supplementary information

Supplementary Information

Description of Additional Supplementary Files

Supplementary Movie 1

Supplementary Movie 2

## Data Availability

The X-ray crystallographic coordinates for structures reported in this study have been deposited at the Cambridge Crystallographic Data Centre (CCDC), under deposition numbers 1859441. These data can be obtained free of charge from The Cambridge Crystallographic Data Centre via www.ccdc.cam.ac.uk/data_request/cif. The authors declare that other data supporting the findings of this study are available from the corresponding author upon reasonable request.
