# Supplementary material for: Stable radical anions generated from a porous perylenediimide metal-organic framework for boosting near-infrared photothermal conversion
Source: Nat Commun. 2019 Feb 15;10:767. doi: 10.1038/s41467-019-08434-4 (PMC6377642; doi:10.1038/s41467-019-08434-4)
Supplement: Supplementary file 1 — Supplementary Information [file 41467_2019_8434_MOESM1_ESM.docx]

**Stable radical anions generated from a porous** **perylenediimide metal-organic framework for boosting** **near-infrared photothermal conversion**

**Baozhong Lü** et al.

**Supplementary Methods**

**Materials and Methods:** All chemical materials were purchased from commercial sources and used without further purification unless particularly pointed out. ^1^H-NMR spectra were recorded on a Bruker 400 (400 MHz ^1^H) spectrometer at room temperature. Matrix-assisted laserdesorption ionization time-of-flight mass spectrometry (MALDI TOF MS) was determined on AXIMA-CFR plus MALDI TOF mass spectrometer. X-ray diffraction (XRD) patterns of the powder were recorded using a Rigaku 2500VB2+PC diffractometer using the Cu Kα radiation (λ=1.541844 Å) at 40 kV and 50 mA with the step-scanned mode in 0.04° (2θ) per step and count time of 10s/step in the range from 5 to 40°. Thermogravimetric analysis (TGA) was conducted on a TGA/SDTA851e thermobalance with a heating rate of 10 ^o^C min^-1^. Fourier transform infrared (FT-IR) spectra were recorded using a Thermo Nicolet Nexus FT-IR devise with the Smart Golden Gate ATR attachment in the range of 4000-500 cm^–1^ with 2 cm^–1^ resolution. Fluorescence spectroscopic studies were performed on a fluorescence spectrophotometer (Horiba JobinYvon FluoroMax-4 NIR, NJ, USA). UV-VIS-NIR spectra were obtained on a spectrometer (UV-3600). The percentage contribution of each lifetime component to the total decay curve, photoluminescence quantum yield (PLQY) and NIR emission spectrum were recorded using an Edinburgh Instruments' FLS 980 fluorescence spectrometer. The surface morphology was investigated by field emission scanning electron microscopy (FESEM, JSM-7500F, JEOL, Japan). Transmission electron microscopy investigations were carried out on a Hitachi HT7700 instrument, TPA adsorption isotherms were measured by a Micrometics 3flex gas sorption analyzer, a sample of 150.6 mg Zr-PDI was pre-activated at 120 ^o^C to remove all residual solvent. TPA adsorption analyses were performed at 25 ^o^C and under vapor pressure of TPA.

**Single crystal measurements:** Single crystal X-ray diffraction measurements were carried out on a Bruker D8 Venture diffractometer outfitted with a PHOTON-100 CMOS detector, using monochromatic microfocus MoKα radiation (λ = 0.71073 Å) that was operated at 50 kV and 40 mA at 153 K by chilled nitrogen flow controlled by a KRYOFLEX II low temperature attachment. Single crystals were selected and mounted on a nylon loop in Paratone-N cryoprotectant. Unit cell determination was performed in the Bruker SMART APEX III software suite. The data sets were reduced and a multi-scan spherical absorption correction was implemented in the SCALE interface and refined with the Shelx2014 software package. CCDC 1859441 contains the supplementary crystallographic data for this paper. These data can be obtained free of charge from the Cambridge Crystallographic Data Centre.

**Electron paramagnetic resonance (EPR):** EPR measurements were performed at X-band (~9.5 GHz) using a Bruker Elexsys E680-X/W EPR spectrometer. The temperature was controlled by a continuous flow cryostat using liquid N_2_.

**Synthesis of P-2COOH:** The ligand N, N’-di-(4-benzoic acid)-1, 2, 6, 7-tetrachloroperylene-3, 4, 9, 10-tetracarboxylic acid diimide (**P-2COOH**) was synthesized according to the literature with some improvement.^1^ Briefly, 1,6,7,8-tetrachloroperylene-3,4,9,10-tetracarboxylic acid dianhydride (1.00 g, 1.89 mmol), aminobenzoic acid (2.59 g, 18.9 mmol), and propionic acid (25 mL) were stirred at 160 ^o^C for 16 h. After the mixture was cooled to room temperature, water was added and the precipitate was filtered and washed with a mixture of methanol/water (1:1) and dried in vacuum to yield an orange solid, then recrystallized the obtained solid with DMF twice, pure red power **P-2COOH** was obtained finally. ^1^H NMR (400 MHz, DMSO) δ 13.20 (s, 1H), 8.62 (s, 2H), 8.14 (d, J = 8.4 Hz, 2H), 7.58 (d, J = 8.4 Hz, 2H). MS-MALDI-TOF (*m z*^-1^): [MH^+^] Calcd. 766.95, found 766.89.

**Supplementary Table 1**. Crystal data and structure refinement for **Zr-PDI**.

| Empirical formula | C_80_H_36_Cl_8_N_4_O_27_Zr_3_ |
| --- | --- |
| Formula weight | 2042.39 |
| Temperature/K | 296.15 |
| Crystal system | tetragonal |
| Space group | I4_1_/a |
| a/Å | 18.085(3) |
| b/Å | 18.085(3) |
| c/Å | 86.656(12) |
| α/° | 90 |
| β/° | 90 |
| γ/° | 90 |
| Volume/Å^3^ | 28343(9) |
| Z | 8 |
| ρ_calc_g/cm^3^ | 0.957 |
| μ/mm^‑1^ | 0.416 |
| F(000) | 8128.0 |
| Crystal size/mm^3^ | 0.15 × 0.12 × 0.11 |
| Radiation | MoKα (λ = 0.71073) |
| 2Θ range for data collection/° | 4.504 to 50.048 |
| Index ranges | -21 ≤ h ≤ 21, -21 ≤ k ≤ 11, -97 ≤ l ≤ 103 |
| Reflections collected | 44548 |
| Independent reflections | 12456 [R_int_= 0.0504, R_sigma_= 0.0556] |
| Data/restraints/parameters | 12456/546/563 |
| Goodness-of-fit on F^2^ | 1.058 |
| Final R indexes [I>=2σ (I)] | R_1_= 0.0882, wR_2_= 0.2577 |
| Final R indexes [all data] | R_1_= 0.1037, wR_2_= 0.2708 |

**
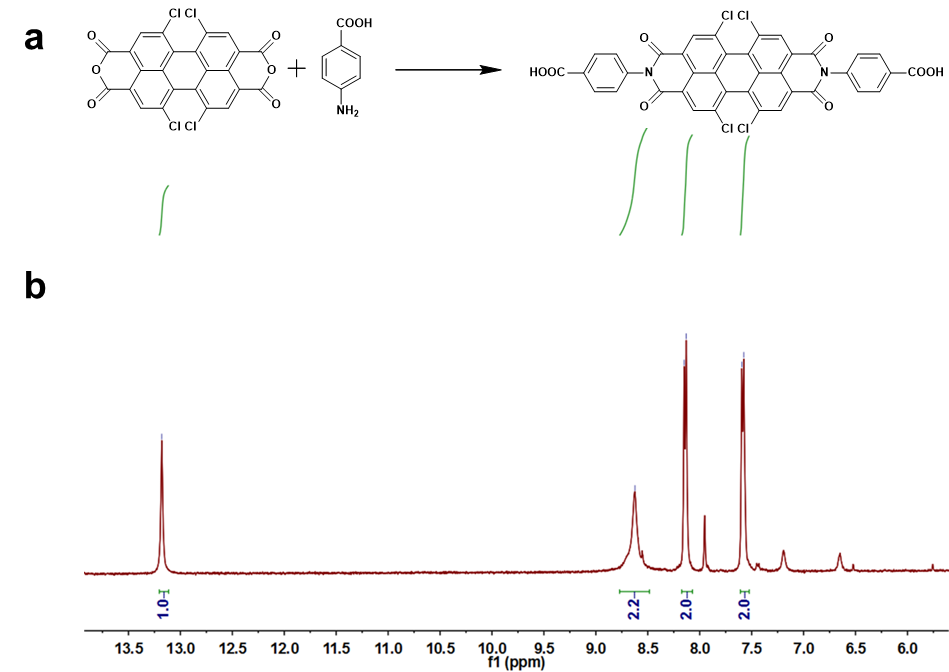
**

**Supplementary** **Figure 1.** Synthetic route (a) and ^1^H NMR spectrum (b) of **P-2COOH**.


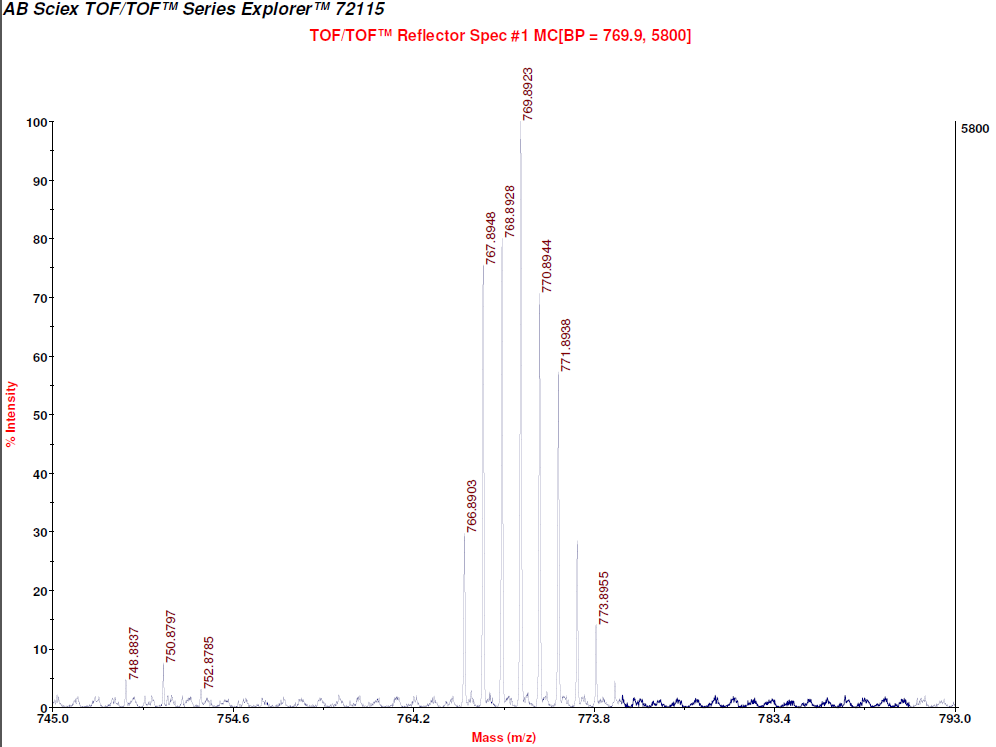


**Supplementary** **Figure 2.** MALDI-TOF-MS of **P-2COOH**.


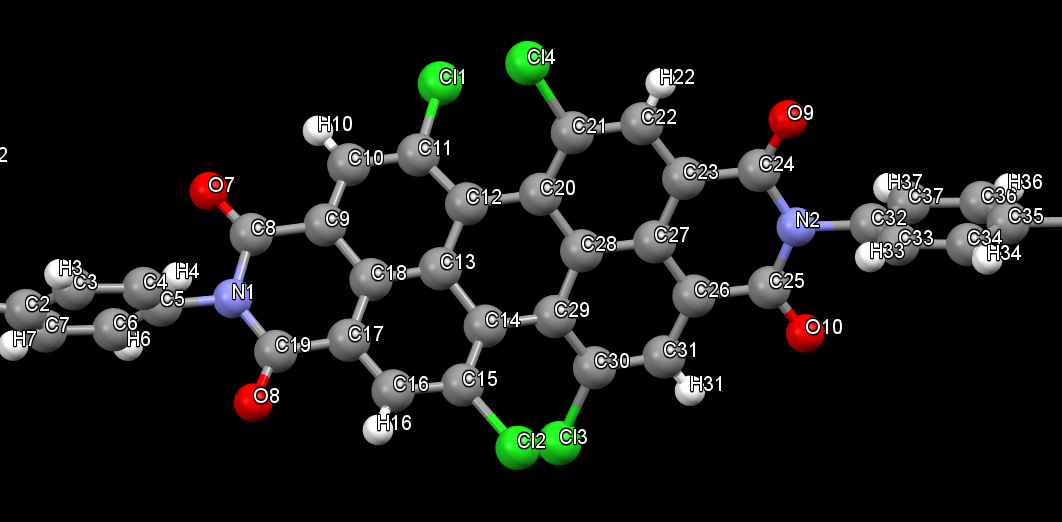


**Supplementary** **Figure 3.** The central six-membered ring is highly twisted, ball-and-stick representation of a dihedral angle of 38.3°.


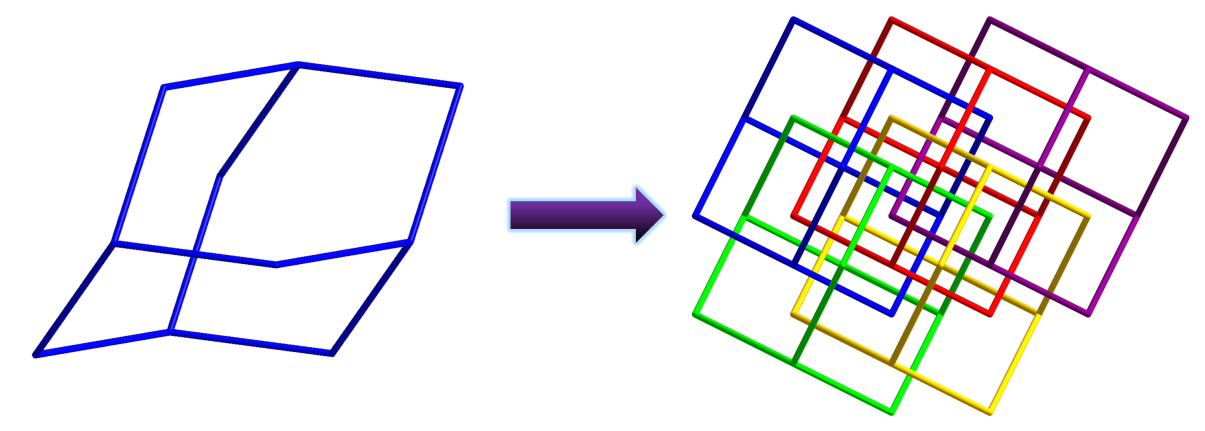


**Supplementary** **Figure 4.** Simplification of single distorted adamantanoid cage in **Zr-PDI** (left) and schematic view of the 5-fold interpenetration along the c-axis (right).

**
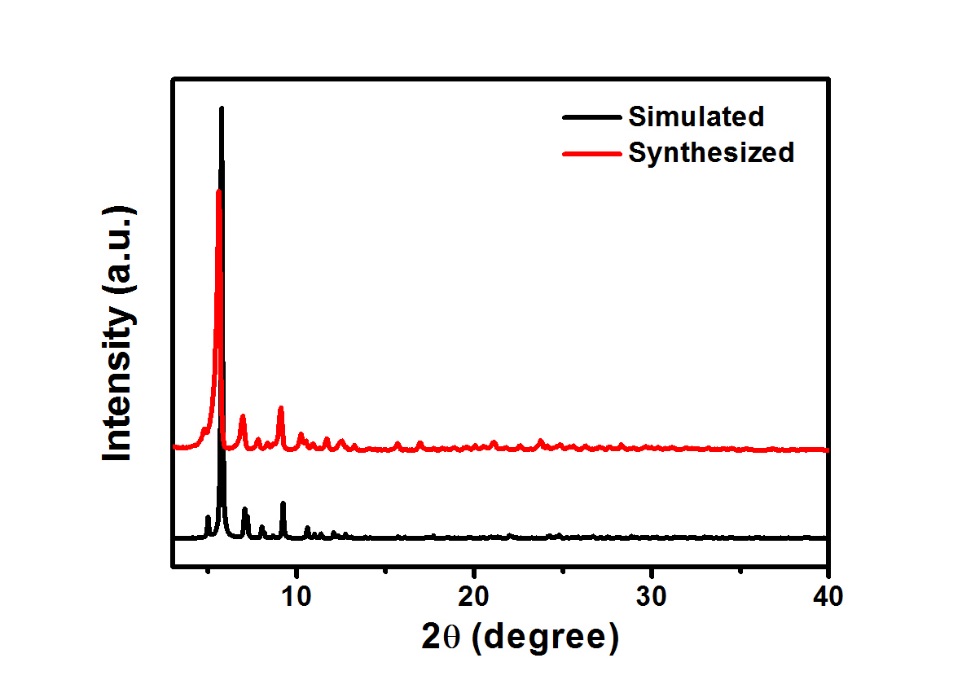
**

**Supplementary** **Figure 5.** PXRD patterns of **Zr-PDI**.


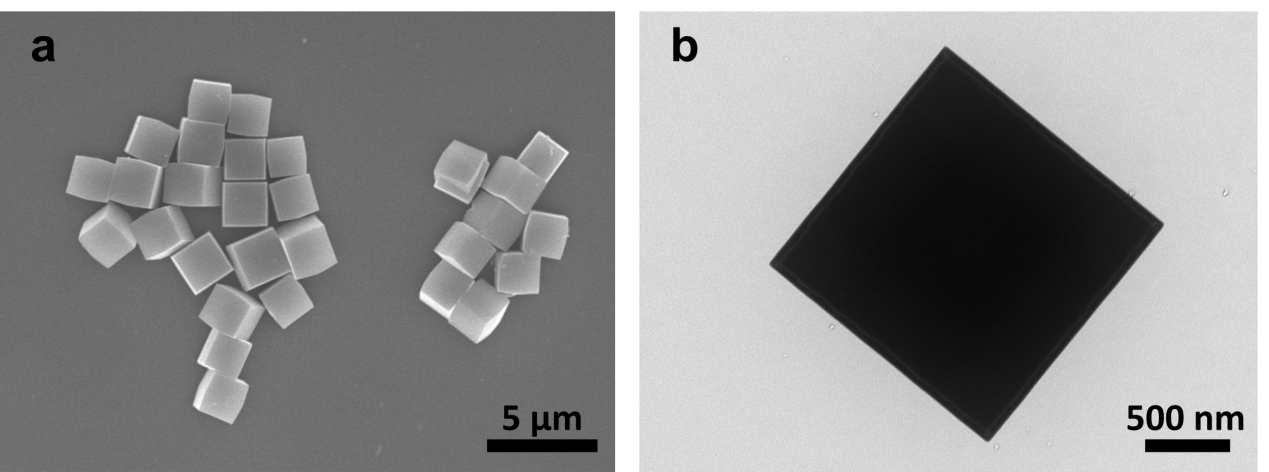


**Supplementary** **Figure 6.** SEM (a) and TEM (b) of **Zr-PDI**.


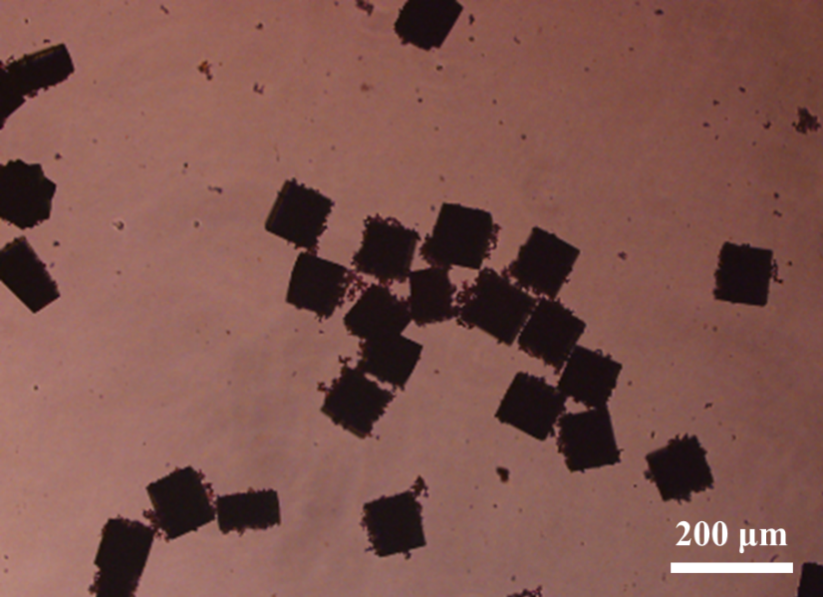


**Supplementary** **Figure 7.** Optical micrograph image of **Zr-PDI** crystals.


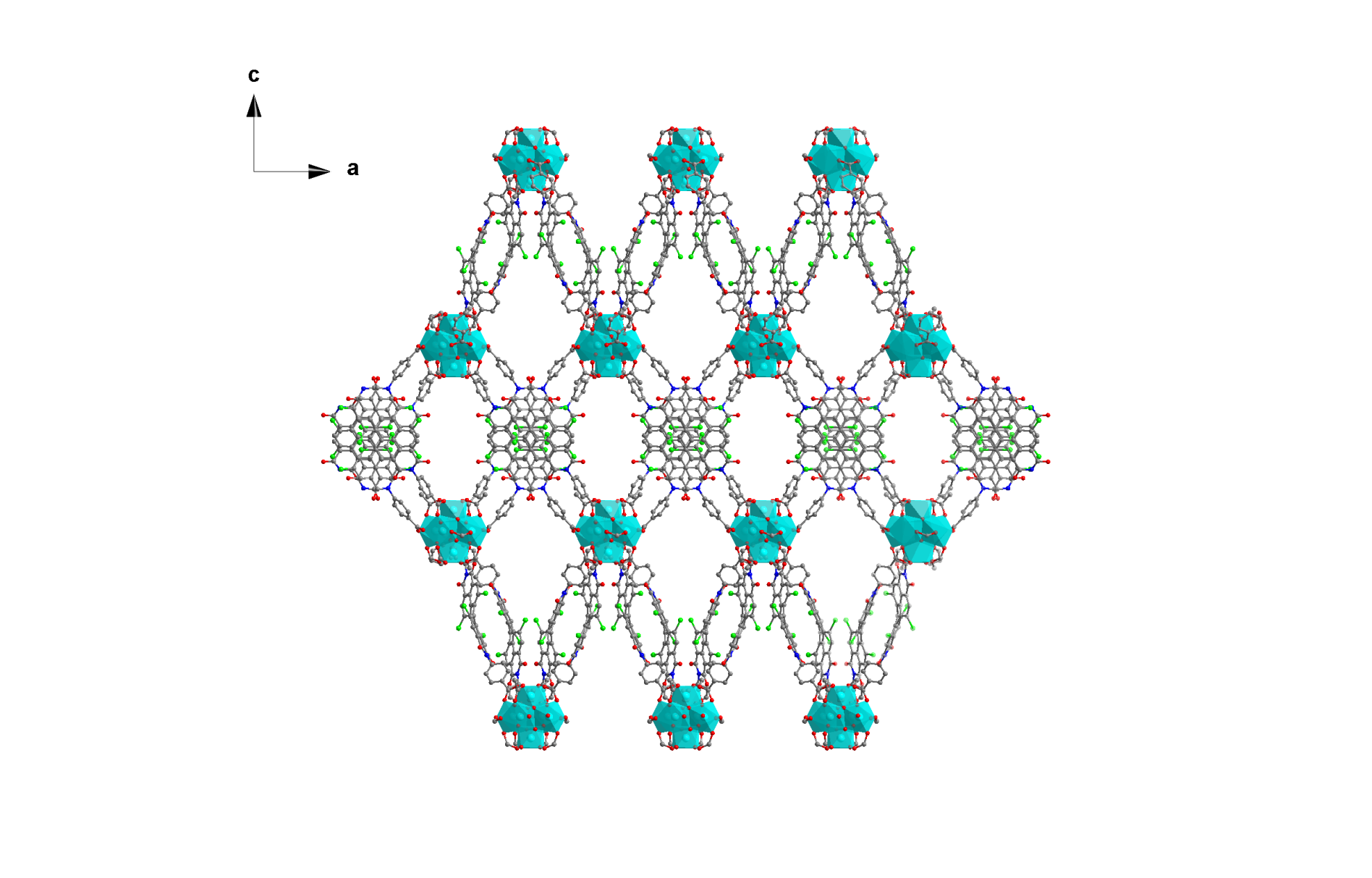


**Supplementary** **Figure 8.** Crystal structure of **Zr-PDI** (viewed from b-axis).


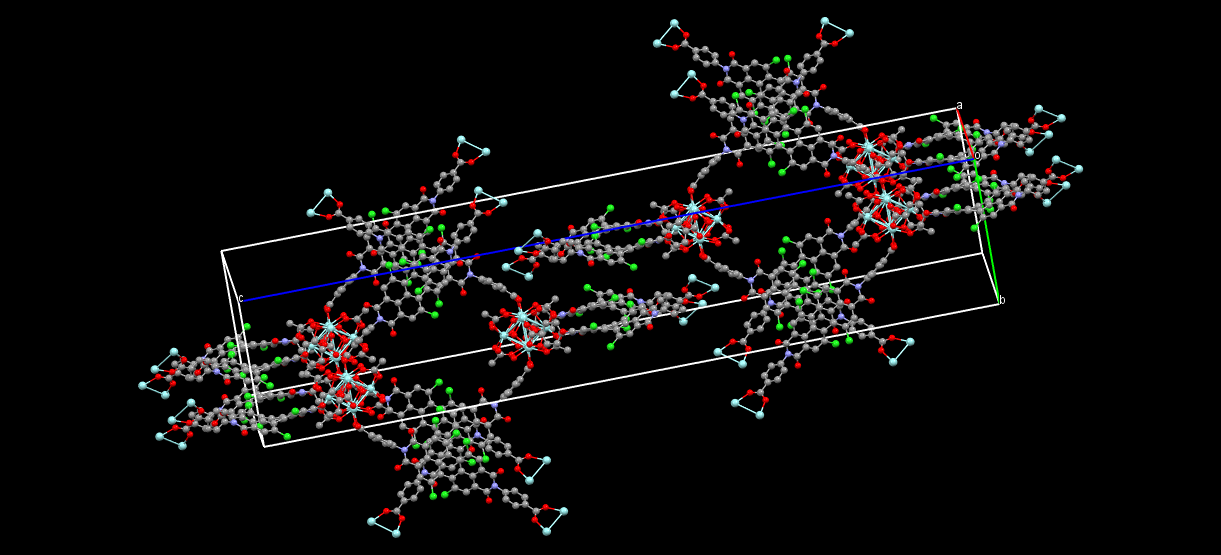


**Supplementary** **Figure 9.** A unit cell of **Zr-PDI**.


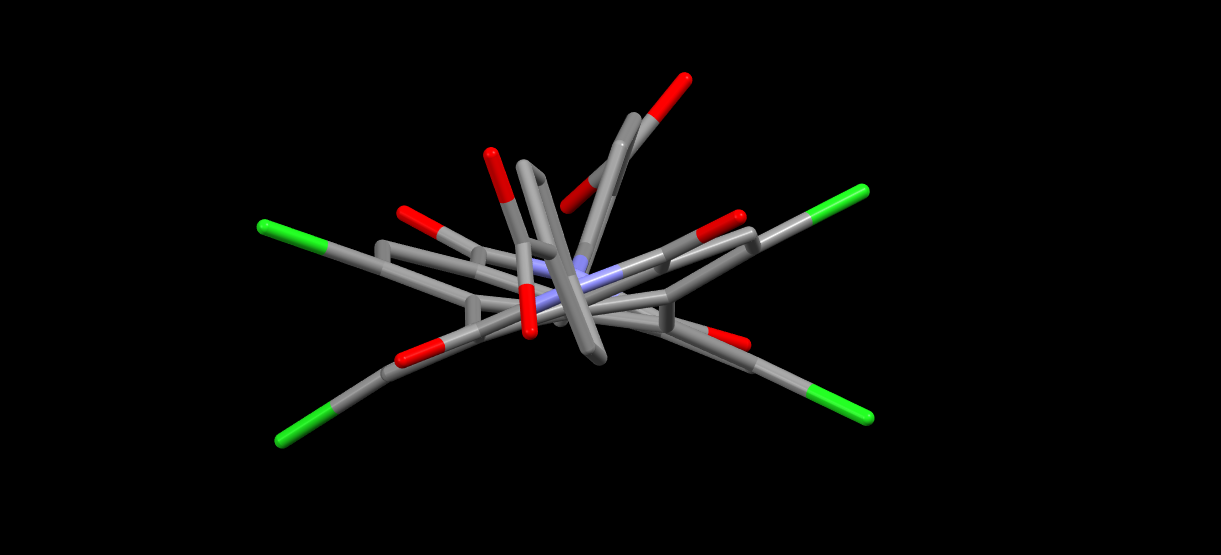


**Supplementary** **Figure 10.** The **P-2COOH** ligand in the **Zr-PDI** framework.


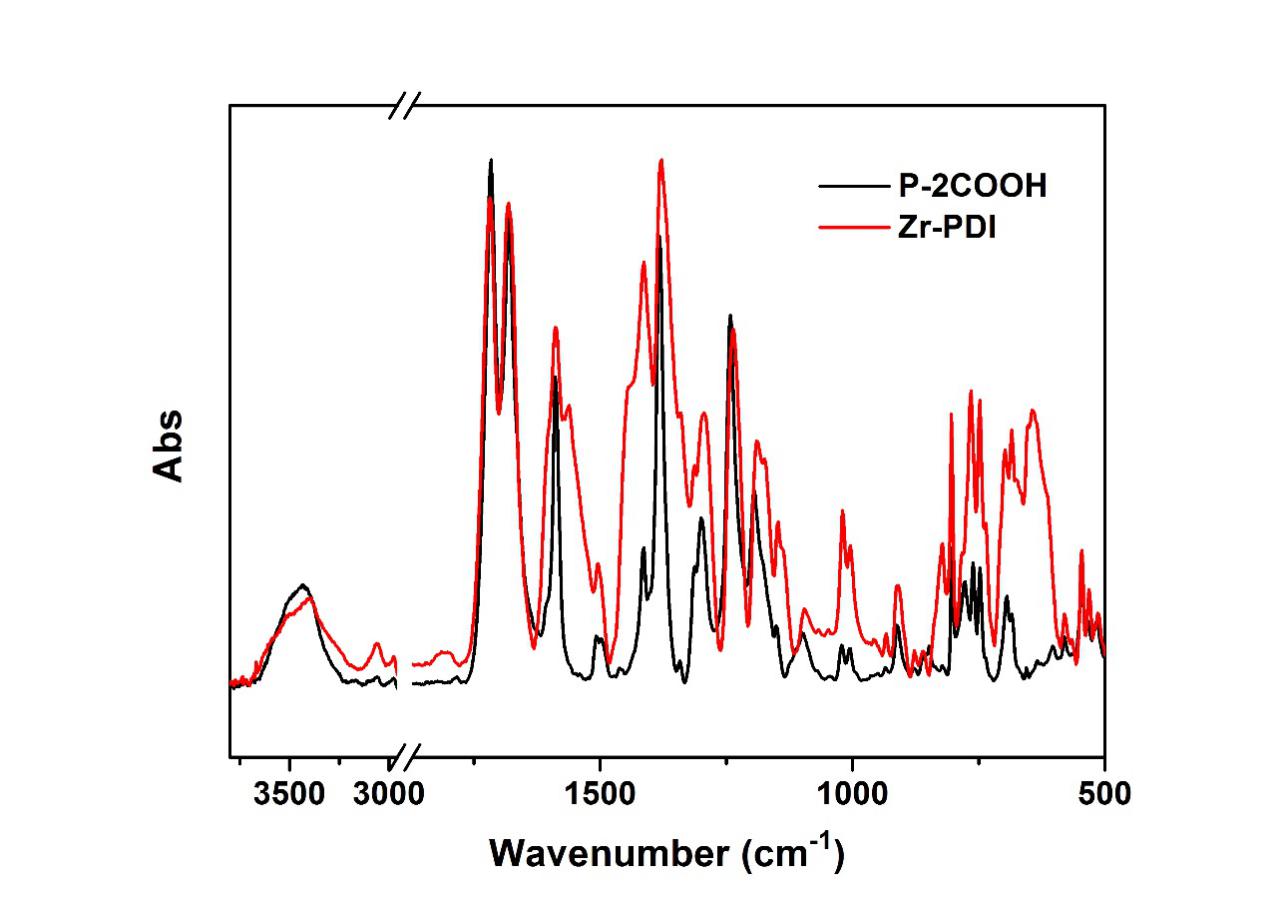


**Supplementary** **Figure 11.** IR spectra of **Zr-PDI** and **P-2COOH**.

**Supplementary Discussion 1**

The incorporation of the PDI linker inside the framework was probed by FTIR, where the bulk powder **Zr-PDI** exhibits vibrational bands similar to the free ligand, although shifted slightly due to coordination with the Lewis acidic Zr^4+^ metal centers.





**Supplementary** **Figure 12.** Thermogravimetric analysis from 40 to 800 ^o^C of **Zr-PDI**.

**Supplementary Discussion 2**

Thermogravimetric analysis measurements show an initial mass loss for **Zr-PDI** at 100-200 ^o^C, which is most likely due to removal of guest solvent molecules (e.g. DMF, H_2_O etc.) from the framework. The material begins to decompose after 350 ^o^C as evidenced by the large mass loss.





**Supplementary** **Figure 13.** The ground-state absorption spectra of the **Zr-PDI** powder and **P-2COOH** in DMF.

**Supplementary Discussion 3**

**P-2COOH** in DMF showed a well-defined vibrational progression typical of PDI derivatives in the monomeric state. The longest wavelength band of **P-2COOH** in DMF (0→0 transition, λ_max_ = 516 nm) was the most intense band, with a second maximum at shorter wavelengths (0→1 transition, λ_max_ = 483 nm) and a shoulder near 425 nm. **Zr-PDI** frameworks has similar absorption spectra to alkyl-substituted PDI thin film.^2^ **Zr-PDI** shows broad, red-shifted absorption in the visible region compared with the spectra of the corresponding monomer in DMF, it’s a result of a combination of excitonic coupling and charge transfer interactions between π-stacked chromophores. NDI-based metal organic framework also has similar phenomenon.^3^





**Supplementary** **Figure 14.** Intense PXRD peak of **P-2COOH** powder.

^

^

**Supplementary** **Figure 15.** UV-Vis-NIR absorption spectra of **P-2COOH** crystalline powder before (black line) and after (red line) treating with TEA and irradiation. **P-2COOH** could only produce small quantity of radical anions with low characteristic absorption peak, which can be attributed to surface adsorption of TEA under irradiation.





**Supplementary** **Figure 16.** Fluorescence spectra of solid **Zr-PDI** and **P-2COOH** in DMF. Broad peak at around 657 nm can be regarded as an excimer emission.


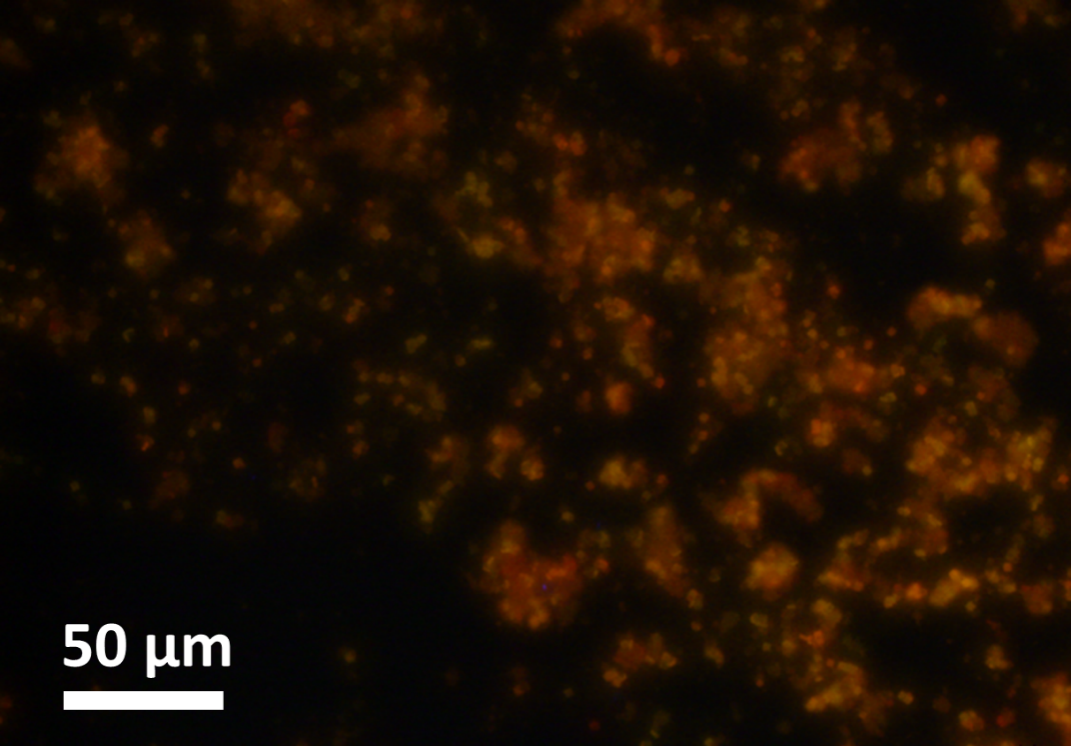


**Supplementary** **Figure 17.** Fluorescence microscope imaging of **Zr-PDI**.


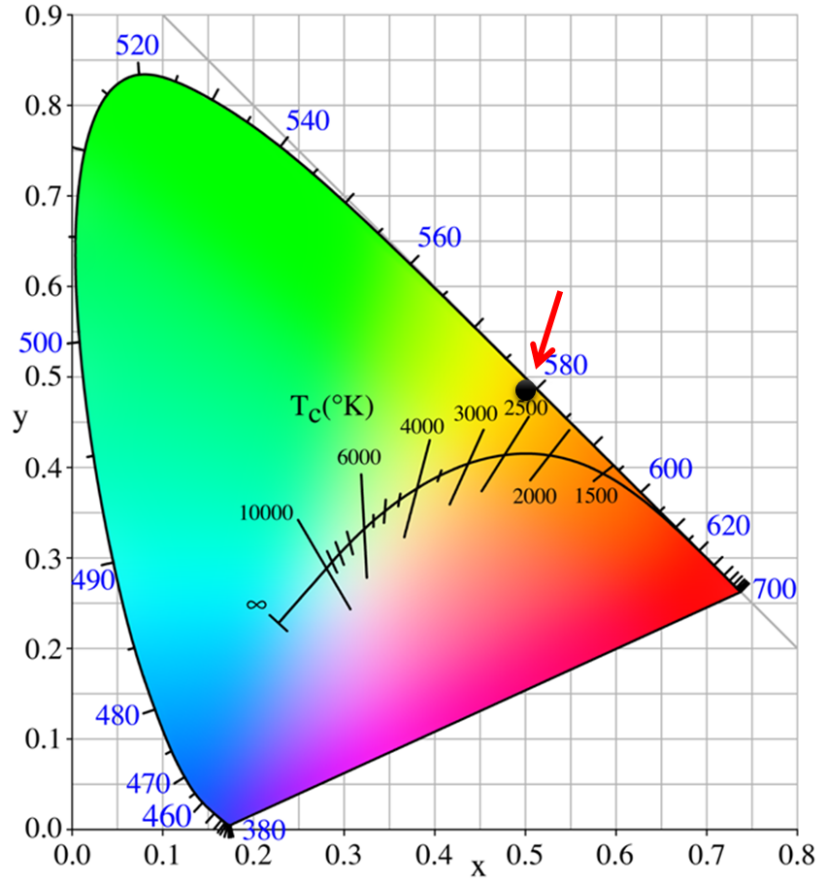


**Supplementary** **Figure 18.** Corresponding Commission Internationale de L’Eclairage (CIE) chromaticity coordinate of **Zr-PDI** was calculated to be (0.50, 0.49).


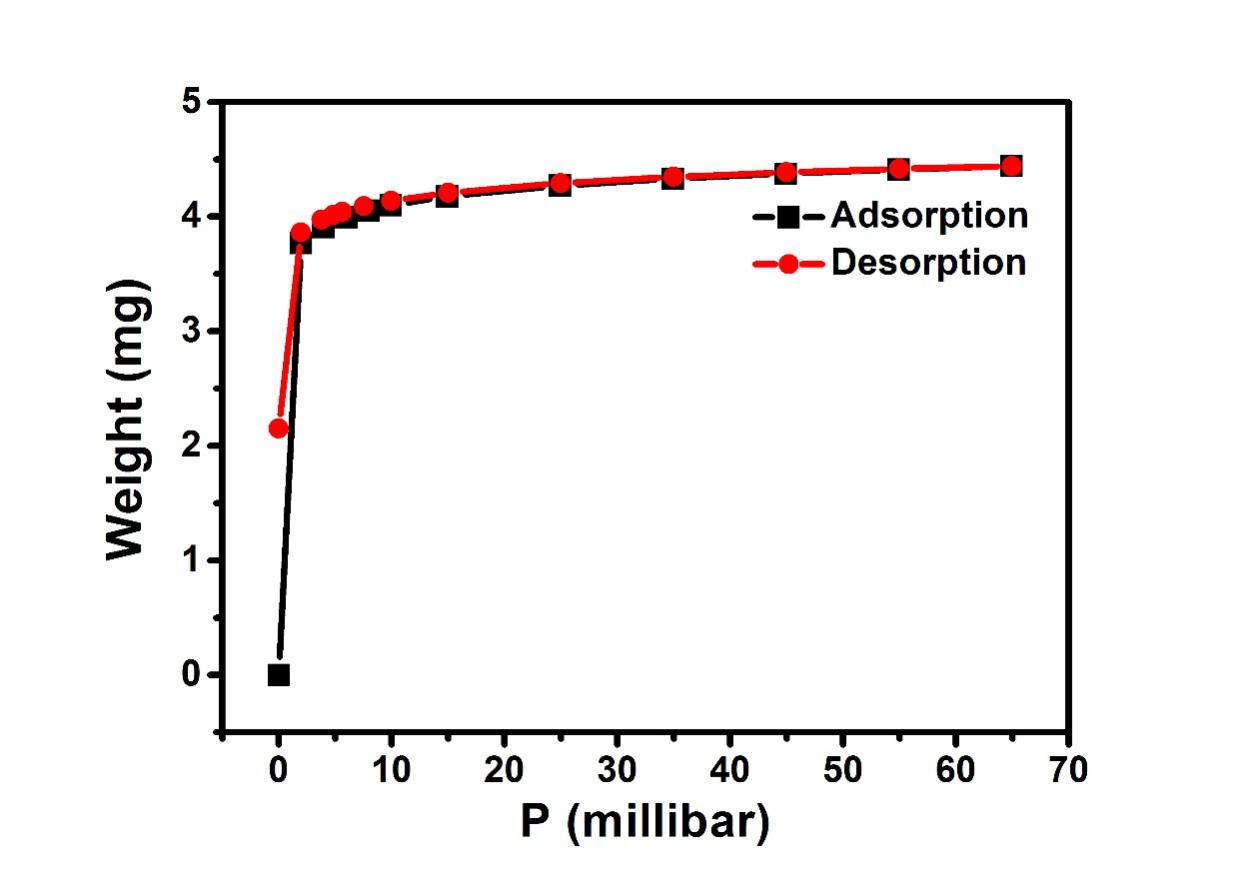


**Supplementary** **Figure 19.** TEA adsorption and desorption measured by a gravimetric method of activated samples of **Zr-PDI**.





**Supplementary** **Figure 20.** Tripropylamine (TPA) adsorption and desorption of activated samples of **Zr-PDI**.


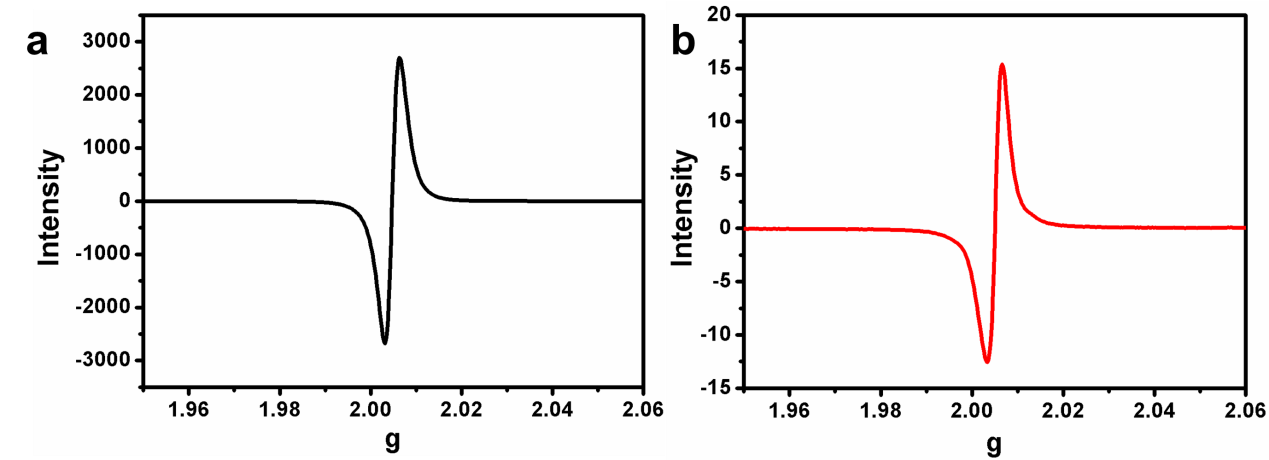


**Supplementary** **Figure 21.** EPR spectra of **Zr-PDI**^•–^ (a) and **P-2COOH**^•–^ (b).





**Supplementary** **Figure 22.** EPR spectrum of **P-2COOH**^•–^ in DMF.


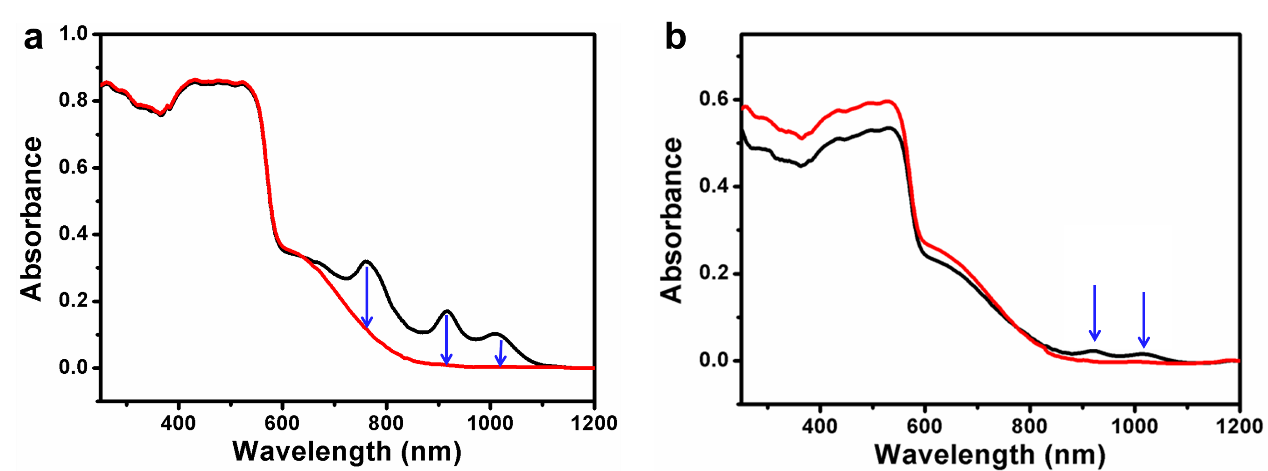


**Supplementary** **Figure 23.** UV-Vis -NIR absorption of radical anions produced by **P-2COOH** (a) or **P-2COOH**/ZrCl_4_ mixture (b). Treating the sample under ambient conditions for 12 h, the characteristic absorption peak of the radical anions will disappear, indicating the instability of radical anions produced by **P-2COOH**.


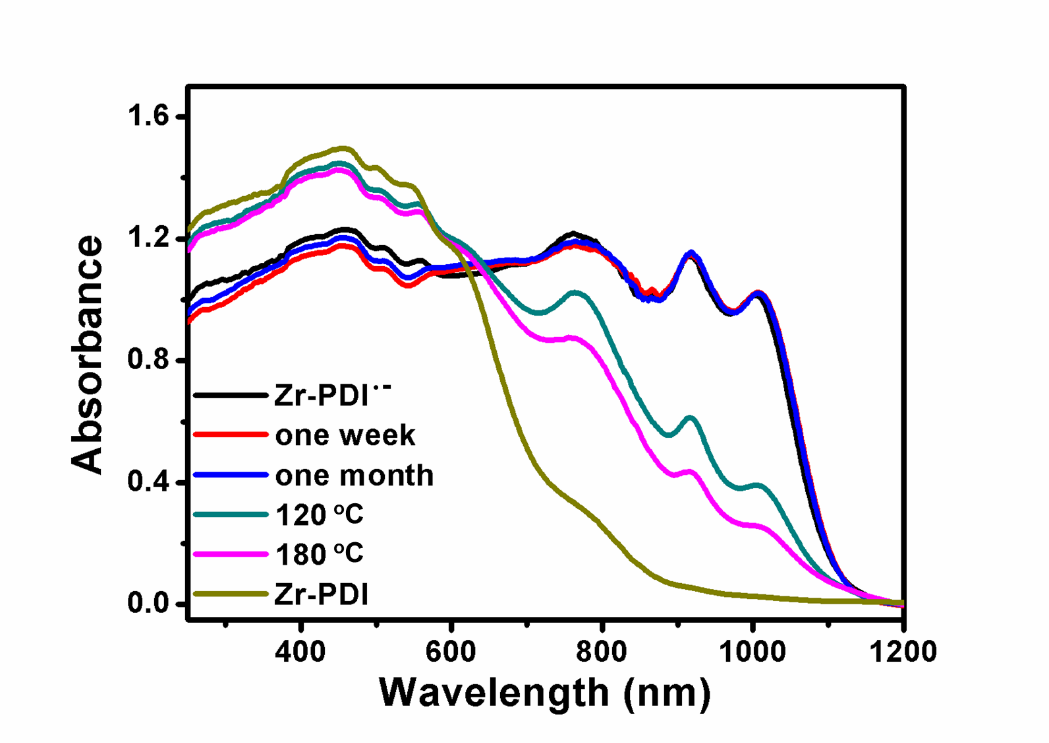


**Supplementary** **Figure 24.** Stability tests, TEA-loaded **Zr-PDI**^•–^ is incubated under various conditions, the **Zr-PDI**^•–^ powders are kept under ambient environment or different temperature for 1 hour prior to measurement.


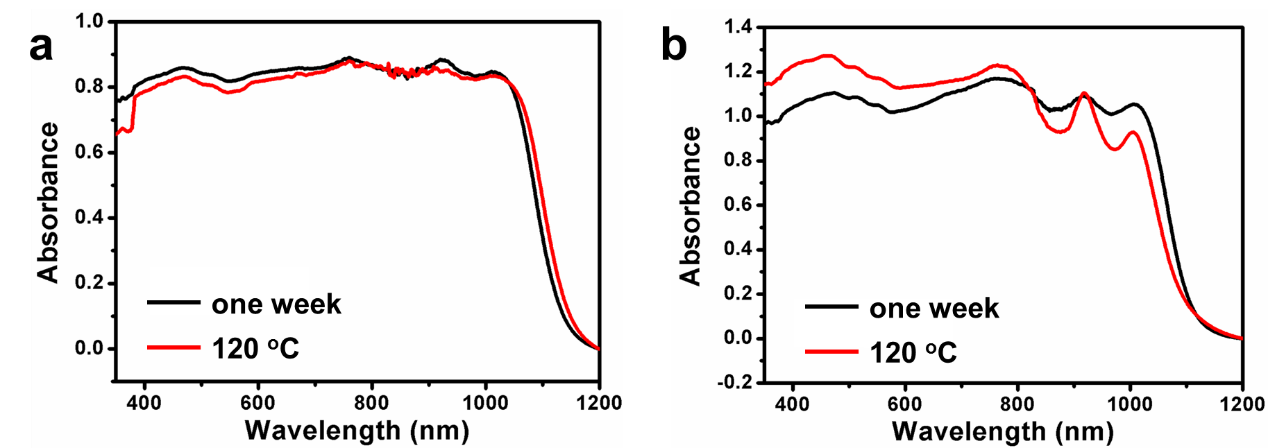


**Supplementary** **Figure 25.** Stability tests, EDA-loaded (a) and TPA-loaded (b) **Zr-PDI**^•–^ is incubated under various conditions, the **Zr-PDI**^•–^ powders are kept under ambient environment for a week or at 120 ^o^C for 1 hour prior to measurement.


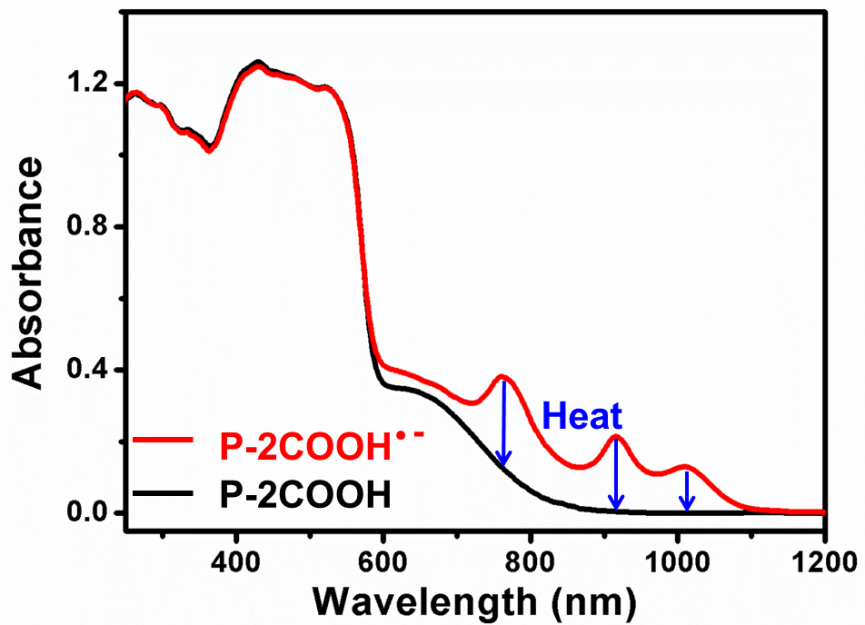


**Supplementary** **Figure 26.** UV-Vis-NIR absorption of **P-2COOH**^•–^. Upon heating of the sample, the characteristic absorption peak of the radical anions will disappear, indicating the instability of radical anions produced by **P-2COOH**.





**Supplementary** **Figure 27.** UV-Vis-NIR absorption of **Zr-PDI** after treatment with different amines, indicating that the large s amines (large than 14 Å) can hardly access the porous **Zr-PDI** and **Zr-PDI**^•–^ cannot be produced.


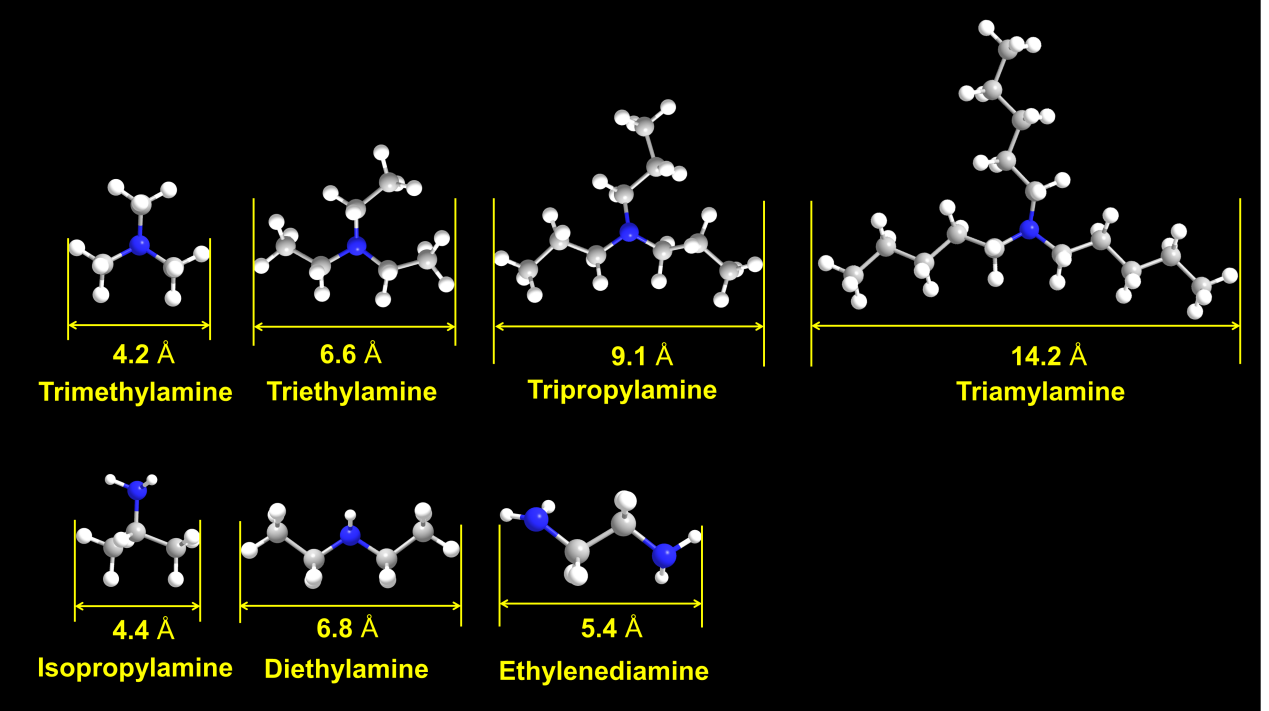


**Supplementary** **Figure 28.** The structures and sizes of different amines calculated using ChemDraw 3D in the lowest energy state. The blue, gray, and white spheres represent nitrogen atom, carbon atom and hydrogen atom, respectively.


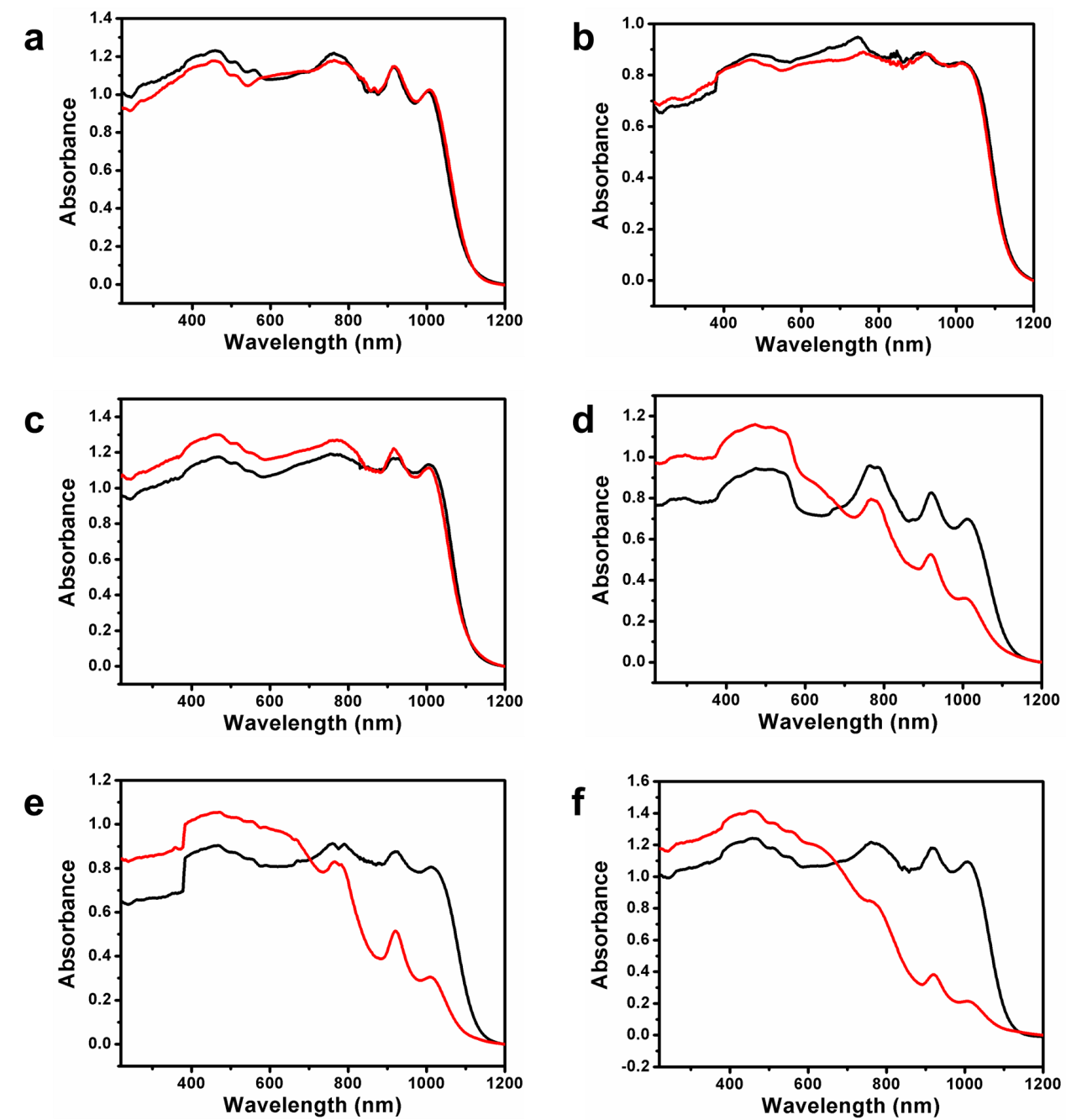


**Supplementary** **Figure 29.** UV-VIS-NIR spectra of **Zr-PDI**^•–^ loading with different amines before (black line) and after (red line) treatment under ambient conditions for a week. Triethylamine (a), Ethylenediamine (b), Tripropylamine (c), Diethylamine (d), Isopropylamine (e), and Trimethylamine (f).

**Supplementary Table 2.** Boiling point of different amines.

| Amine | Trimethylamine | Triethylamine | Tripropylamine | Triamylamine |
| --- | --- | --- | --- | --- |
| Boiling point (^o^C) | ~ 3 | ~ 90 | ~ 155 | ~ 243 |
| Amine | Isopropylamine | Diethylamine | Ethylenediamine |  |
| Boiling point (^o^C) | ~ 33 | ~ 55 | ~ 116 |  |


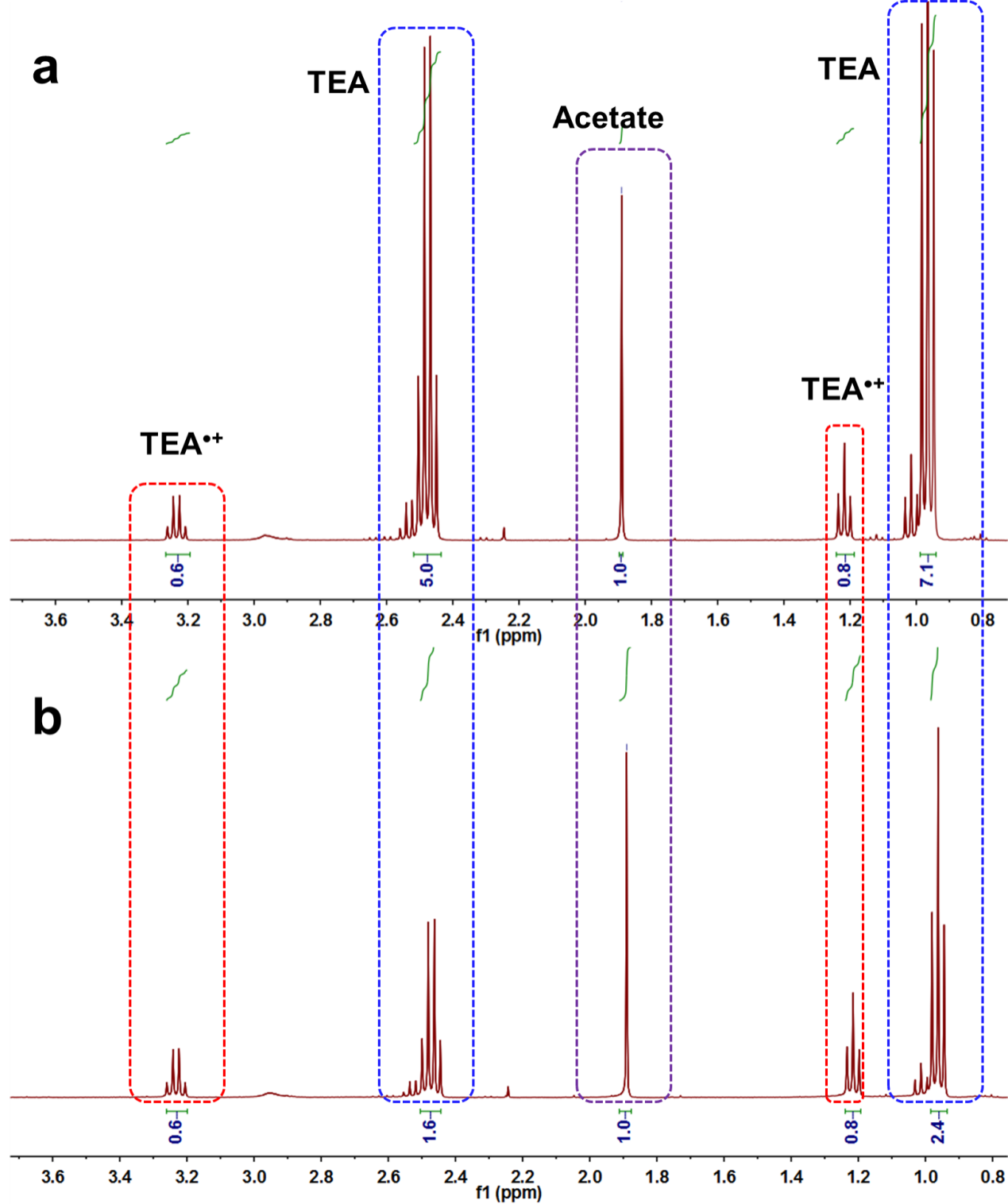


**Supplementary** **Figure 30.** ^1^H NMR spectra of alkaline-digested Zr-PDI after treatment with TEA vapor and irradiation with blue light (455 nm, 10 minutes) (a), then kept at 120 ^o^C for an hour (b). Due to the paramagnetic nature of **Zr-PDI**^•–^, only strongly broadened resonances due to PDI were detected by proton NMR spectroscopy. The -CH_3_ integration of acetate in **Zr-PDI** was used as reference substance, the amount of TEA decreased, but the amount of TEA^•+^ produced by the PET process remained almost constant, the stability will similarly remain after placed the treated **Zr-PDI**^•–^ under ambient conditions for another month, indicating the stability is irrelevant to the loading amount of TEA.





**Supplementary** **Figure 31.** UV-Vis-NIR absorption of **Zr-PDI**^•–^ kept at 120 ^o^C for an hour (black line) and then placed at ambient conditions for one month (red line).


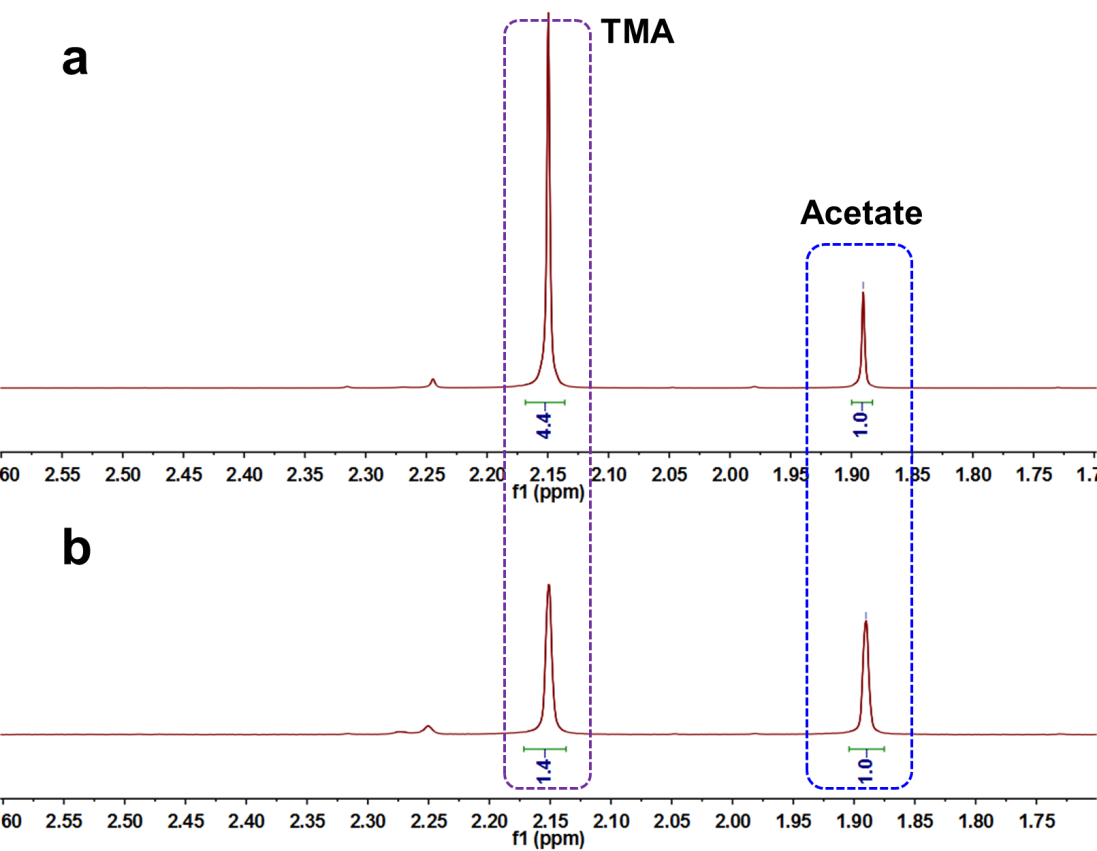


**Supplementary** **Figure 32.** ^1^H NMR spectra of alkaline-digested Zr-PDI after treatment with trimethylamine (TMA) vapor and irradiation with blue light (455 nm, 10 minutes) (a), the sample was then kept at ambient conditions for one week (b). The -CH_3_ integration of acetate in **Zr-PDI** was used as reference substance, the amount of TMA decreased by 68.2%, indicating the instability of **Zr-PDI**^•–^ when using TMA as electron donor under ambient conditions.


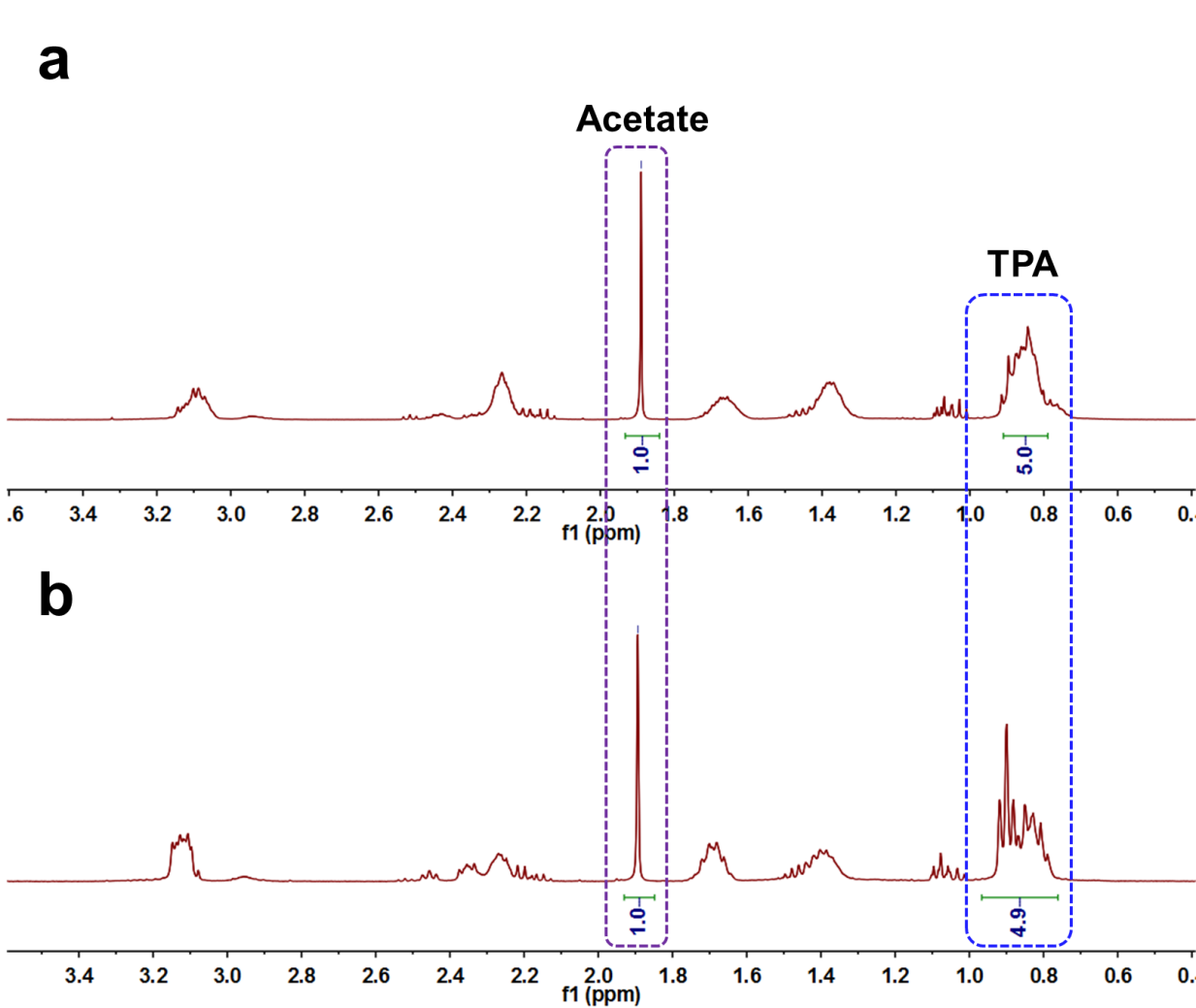


**Supplementary** **Figure 33.** ^1^H NMR spectra of alkaline-digested **Zr-PDI** after reatment with tripropylamine (TPA) vapor and irradiation with blue light (455 nm, 10 minutes) (a), the sample was then kept at ambient condition for one week (b). The -CH_3_ integration of acetate in **Zr-PDI** was used as reference substance, the amount of TPA remained almost unchanged with a retention rate of 98%, indicating the stability of **Zr-PDI**^•–^ using TPA as electron donor.


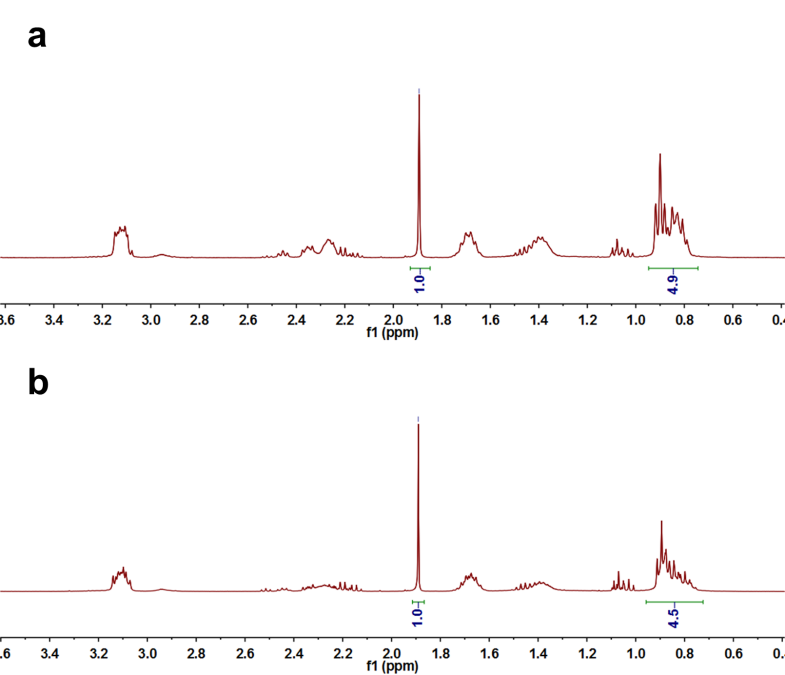


**Supplementary** **Figure 34.** ^1^H NMR spectra of alkaline-digested Zr-PDI after treatment with TPA vapor and irradiation with blue light (455 nm, 10 minutes) (a) and kept at about 120 ^o^C for an hour (b). The amount of TPA decreased slightly, indicating that amines with high boiling points remain in **Zr-PDI** even at high temperatures.


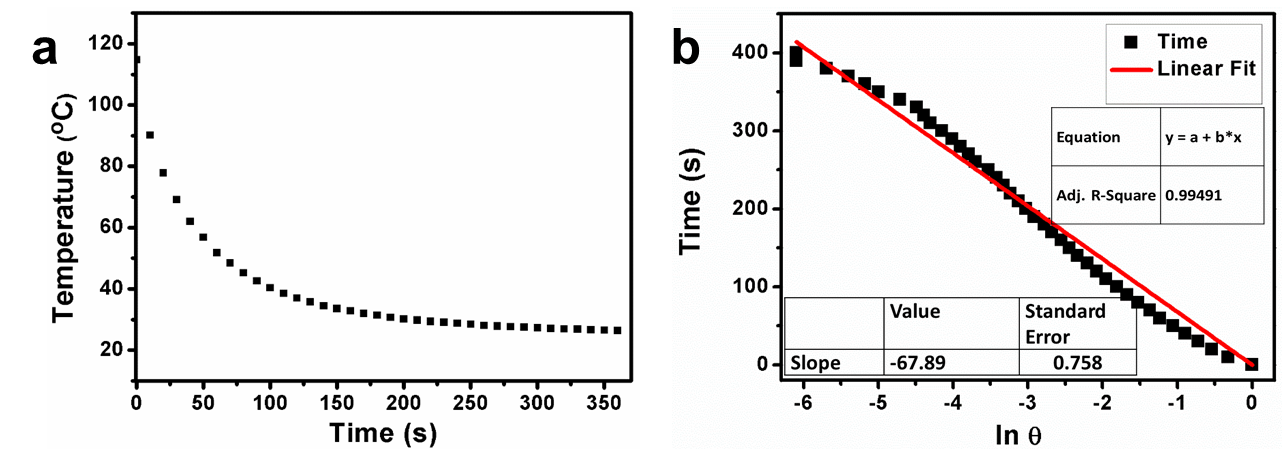


**Supplementary** **Figure 35.** The cooling curve of **Zr-PDI** film after irradiation with 808 nm laser (0.7 W cm^-2^) (a) and its corresponding time-ln*θ* linear curve (b).

**Supplementary Note 1**

The conversion efficiency was determined according to previous method.^4^ Details are as follows:

Based on the total energy balance for this system:

$$\sum_{i} m_{i}C_{pi}\frac{ⅆT}{ⅆt}=Q_{s}-Q_{loss}$$

where *m_i_* (0.32 g) and *C_p,i_* (0.8 J (g ^o^C)^-1^ ) are the mass and heat capacity of system components (Zr-PDI samples and quartz glass), respectively. *Q_s_* is the photothermal heat energy input by irradiating NIR laser to Zr-PDI samples, and *Q_loss_* is thermal energy lost to the surroundings. When the temperature is maximum, the system is in balance.

*Q_s =_ Q_loss =_ hSΔT_max_*

where *h* is heat transfer coefficient, *S* is the surface area of the container, *ΔT_max_* is the maximum temperature change. The photothermal conversion efficiency *η* is calculated from the following eaquation:

$$\eta=\frac{hS{\Delta T}_{max}}{I\left( 1-{10}^{-A_{808}} \right)}$$

where *I* is the laser power (0.7 W cm^-2^) and *A_808_* is the absorbance of the samples at the wavelength of 808 nm (1.12).

In order to obtain the *hS*, a dimensionless driving force temperature, *θ* is introduced as follows:

$$\theta=\frac{T-T_{surr}}{T_{max}-T_{surr}}$$

where *T* is the temperature of Zr-PDI, *T_max_* is the maximum system temperature (114.7 ^o^C), and *T_surr_* is the initial temperature (25.4 ^o^C).

The sample system time constant *τ_s_*

$$\tau_{s}=\frac{\sum_{ⅈ} m_{i}C_{p,i}}{hS}$$

thus $\frac{ⅆ\theta}{ⅆt}=\frac{1}{\tau_{s}}\frac{Q_{s}}{hS\Delta T_{max}}$ - $\frac{\theta}{\tau_{s}}$

when the laser is off, *Q_s_* = 0, therefore $\frac{ⅆ\theta}{dt}=-\frac{\theta}{\tau_{s}}$ , and $t=-\tau_{s}\ln\theta$

so *hS* could be calculated from the slope of cooling time vs ln$\theta$. Therefore, *τ_s_* is 67.9 s (Supplementary Figure 35) and the photothermal conversion efficiency *η* is 52.3%.

**Supplementary Table 3.** Comparison of the photothermal efficiency (η_PT_) among various materials.

| **Contrast Sample** | **Laser power (W)** | **η_PT_ (%)** | **Classification** | **Ref.** |
| --- | --- | --- | --- | --- |
| Au nanoshells | 2 | 13 | Inorganic materials | 5 |
| Au nanorods | 2 | 21 |  | 5 |
| Cu_9_S_5_ nanocrystals | 0.51 | 25.7 |  | 6 |
| Selenophene derivative polymer films | 2 | 40 | Organic materials | 4 |
| Organic cocrystal | 0.7 | 18.8 |  | 7 |
| Terrylenediimide poly(acrylic acid) | 1 | 41 |  | 8 |
| PDI supramolecular free radicals | 1 | 31.6 | PDI-based radicals | 9 |
| PDI-[glycine-aspartic acid] radicals | 1 | 8.4 (0.02 mM)^a^  16.4 (0.15 mM)^a^  36.8 (1 mM)^a^  59.5 (10 mM)^a^ |  | 10 |
| UiO-66@PAN | 1.5 | 21.6 | MOF-based hybrid materials | 11 |
| ZIF-8@SiO_2_ carbon nanospheres | 1 | 33 |  | 12 |
| PDI MOF (**Zr-PDI**^•–^) | 0.7 | 52.3 | PDI-based MOF radicals | This work |

^a^ Photothermal conversion efficiency with different concentrations.


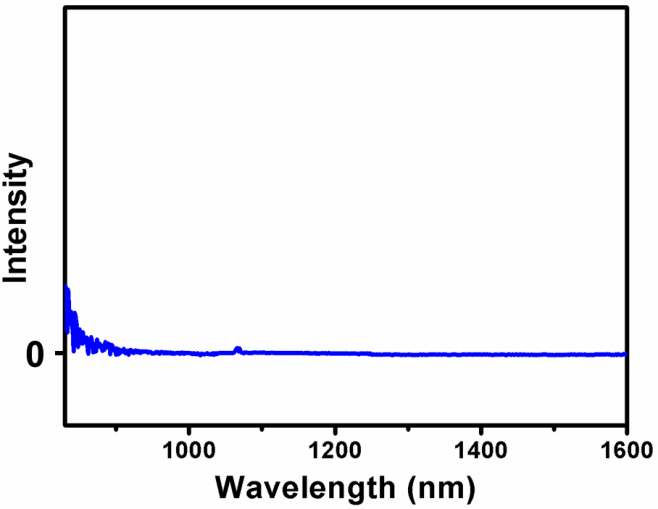


**Supplementary** **Figure 36.** NIR emission spectrum of **Zr-PDI**^•–^ upon irradiation at 808 nm.





**Supplementary** **Figure 37.** N_2_ sorption isotherm of **Zr-PDI** at 77 K after TEA inclusion and photothermal conversion.


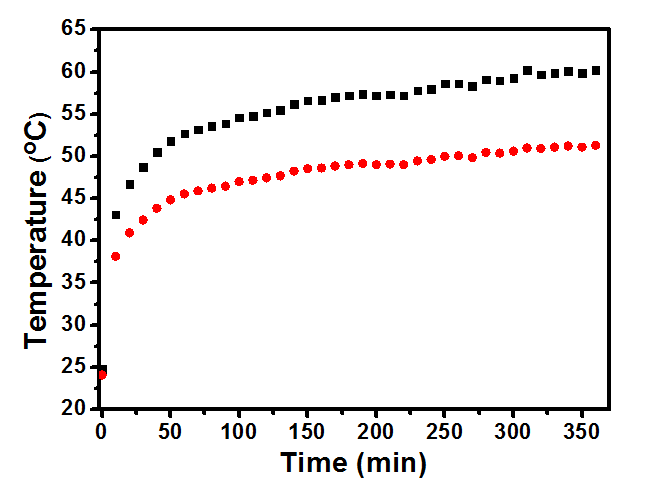


**Supplementary** **Figure 38.** Photothermal conversion curves of quartz glass with **P-2COOH**^•–^ film under 808 nm laser irradiation (0.7 W cm^-2^) in the first test (black) and the second test (red).


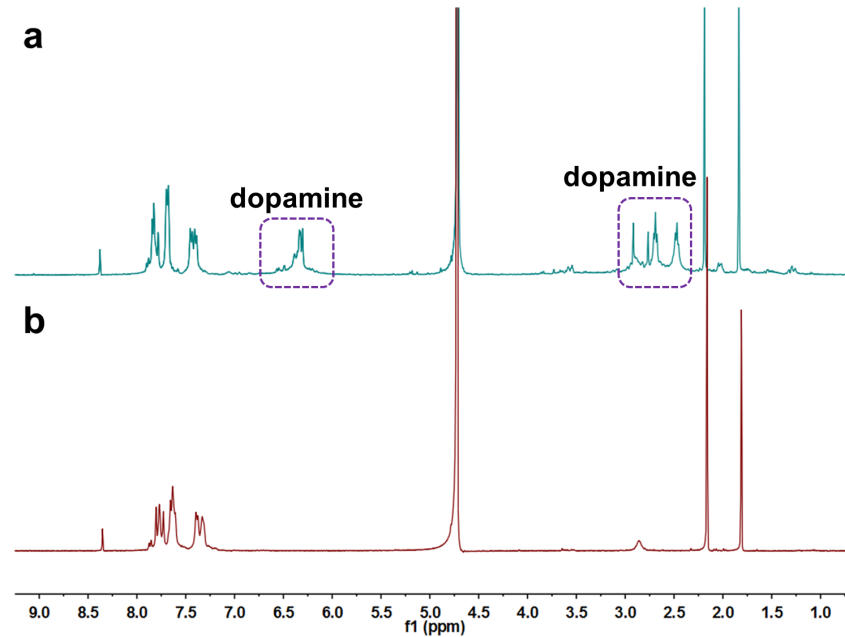


**Supplementary** **Figure 39.** ^1^H NMR spectra of alkaline-digested **Zr-PDI** treated with dopamine (a) and original **Zr-PDI** (b). The additional chemical shifts corresponding to the proton signals of dopamine are observed.





**Supplementary** **Figure 40.** UV-Vis-NIR absorption spectra of **Zr-PDI** crystalline powder before (black line) and after (red line) treatment with dopamine and irradiation. Thus, radical anions can also be produced when using dopamine.

**Supplementary References**

1. Addicott, C., Oesterling, I., Yamamoto, T., Müllen, K. & Stang, P. J. Synthesis of a bis(pyridyl)-substituted perylene diimide ligand and incorporation into a supramolecular rhomboid and rectangle via coordination driven self-Assembly. *J. Org. Chem.* **70**, 797-801 (2005).

2. Hartnett, P. E. et al. Long-lived charge carrier generation in ordered films of a covalent perylenediimide-diketopyrrolopyrrole-perylenediimide molecule. *Chem. Sci.* **6**, 402-411 (2015).

3. McCarthy, B. D., Hontz, E. R., Yost, S. R., Van Voorhis, T. & Dincă, M. Charge transfer or J-Coupling? assignment of an unexpected red-shifted absorption band in a naphthalenediimide-based metal–organic framework. *J. Phys. Chem. Lett.* **4**, 453-458 (2013).

4. Kim, B., Shin, H., Park, T., Lim, H. & Kim, E. NIR-sensitive poly(3,4-ethylenedioxyselenophene) derivatives for transparent photo-thermo-electric converters. *Adv. Mater.* **25**, 5483-5489 (2013).

5. Hessel, C. M. *et al.* Copper selenide nanocrystals for photothermal therapy. *Nano Lett.*, **11**, 2560-2566 (2011).

6. Tian, Q. *et al.* Hydrophilic Cu_9_S_5_ nanocrystals: a photothermal agent with a 25.7% heat conversion efficiency for photothermal ablation of cancer cells *in vivo.* *ACS Nano*, **5**, 9761-9771 (2011).

7. Wang, Y. *et al.* Cocrystals strategy towards materials for near-infrared photothermal conversion and imaging. *Angew. Chem. Int. Ed.* **57**, 3963-3967 (2018).

8. Zhang, S. *et al.* Terrylenediimide-based intrinsic theranostic nanomedicines with high photothermal conversion efficiency for photoacoustic imaging-guided cancer therapy. *ACS Nano*, **11**, 3797-3805 (2017)

9. Jiao, Y., Liu, K., Wang, G., Wang, Y. & Zhang, X. Supramolecular free radicals: near-infrared organic materials with enhanced photothermal conversion. *Chem. Sci.* **6**, 3975-3980 (2015).

10. Cui, L. *et al.* Regulating morphologies and near-infrared photothermal conversion of perylene bisimide via sequence-dependent peptide self-assembly. *Chem. Commun.* **54**, 2208-2211 (2018).

11. Wang, W. *et al.* Nanoscale polymer metal-organic dramework hybrids for effective photothermal therapy of colon cancers. *Adv. Mater.* **28**, 9320-9325 (2016).

12. Wang, S. *et al.* Metal–organic-framework-derived mesoporous carbon nanospheres containing porphyrin-like metal centers for conformal phototherapy. *Adv. Mater.* **28**, 8379-8387 (2016).
