# Supplementary material for: Stable radical anions generated from a porous perylenediimide metal-organic framework for boosting near-infrared photothermal conversion
Source: Nat Commun. 2019 Feb 15;10:767. doi: 10.1038/s41467-019-08434-4 (PMC6377642; doi:10.1038/s41467-019-08434-4)
Supplement: Supplementary file 2 — Description of Additional Supplementary Files [file 41467_2019_8434_MOESM2_ESM.docx]

**Description of Additional Supplementary Files**

**File Name:** Supplementary Movie 1

**Description:** The temperature of Zr-PDI•– powder reached 160 o C in 10 s, under irradiation with NIR laser (808 nm, 0.7 W cm-2).

**File Name:** Supplementary Movie 2

**Description:** Zr-PDI•– powder was fixed on a piece of quartz glass to form a uniform film (0.8 × 0.8 cm, 320 mg). Under irradiation with 808 nm laser light (0.7 W cm2 ), the temperature of the quartz glass was sharply increased to as high as 114 o C from room temperature, i.e, a temperature rise of 89 o C within 200 s.
